# Pharmacokinetic Parameters and Tissue Withdrawal Intervals for Sheep Administered Multiple Oral Doses of Meloxicam

**DOI:** 10.3390/ani11102797

**Published:** 2021-09-25

**Authors:** Sarah Depenbrock, Tara Urbano, Jessie Ziegler, Scott Wetzlich, Maaike O. Clapham, Lisa A. Tell

**Affiliations:** 1 Department of Veterinary Medicine and Epidemiology, School of Veterinary Medicine, University of California, Davis, Davis, CA 95616, USA; sewetzlich@ucdavis.edu (S.W.); moclapham@ucdavis.edu (M.O.C.); latell@ucdavis.edu (L.A.T.); 2 Veterinary Medical Teaching Hospital, School of Veterinary Medicine, University of California, Davis, Davis, CA 95616, USA; tmurbano@ucdavis.edu (T.U.); jcziegler@ucdavis.edu (J.Z.)

**Keywords:** residue, slaughter, anti-inflammatory, NSAID, ovine

## Abstract

**Simple Summary:**

Meloxicam is an anti-inflammatory drug used to treat pain and inflammation in ruminants including sheep, and pharmacokinetic studies are needed to protect the food supply from drug residues after use in food-producing animals. This study estimated plasma pharmacokinetic parameters and meat withdrawal intervals (WDI) for market sheep after multiple daily oral doses of meloxicam. Single and multiple dose plasma pharmacokinetic studies, a multi-dose tissue depletion study, and a follow-up study to investigate if events prior to slaughter were associated with differences in plasma meloxicam concentrations, all using sample data collected after completion of dosing, were completed. Using regulatory agency methods for calculating withdrawal times, an estimated WDI of at least 10 d following the last dose is recommended for market lambs treated with ten daily oral 1 mg/kg doses of meloxicam tablets suspended in water. The effect of events surrounding slaughter on plasma meloxicam concentrations in lambs is unknown but should be considered if plasma samples are obtained immediately prior to or during the slaughter process and used for pharmacokinetic investigations.

**Abstract:**

Meloxicam is an anti-inflammatory drug used to treat pain and inflammation in ruminants including sheep, and pharmacokinetic studies are needed to protect the food supply from drug residues after use in food-producing animals. This study estimated plasma pharmacokinetic parameters and meat withdrawal intervals (WDI) for market sheep after multiple daily oral doses of meloxicam. Single and multiple dose plasma pharmacokinetic studies, a multi-dose tissue depletion study, and a follow-up study to investigate if events prior to slaughter were associated with differences in plasma meloxicam concentrations, all using sample data collected after completion of dosing, were completed. Using regulatory agency methods for calculating withdrawal times, an estimated WDI of at least 10 d following the last dose is recommended for market lambs treated with 10 daily oral 1 mg/kg doses of meloxicam tablets suspended in water. The effect of events surrounding slaughter on plasma meloxicam concentrations in lambs is unknown but should be considered if plasma samples are obtained immediately prior to or during the slaughter process and used for pharmacokinetic investigations.

## 1. Introduction

Pain and inflammation can be caused by disease states or some routine management practices such as castration or tail docking in sheep. Meloxicam is a non-steroidal anti-inflammatory drug that has been used to treat pain and inflammation in ruminants including sheep, and has gained increased popularity in the past decade [1,2,3]. Oral meloxicam therapy allows for convenient dosing that can be accomplished on farm without equipment and training necessary to give injections. Suggested therapeutic plasma concentrations for other species have been reported between 0.13–0.2 µg/mL in humans [4] to 0.57–0.93 µg/mL in horses [5] and at least 1 µg/mL in sheep [1]. A study investigating lameness in sheep demonstrated that a single 1.0 mg/kg IV dose alleviated some experimentally induced signs of inflammation, however the associated plasma meloxicam concentration relative to the effective dose in 50% of the study population (ED50) was not reported [2]. Pharmacokinetic parameters for sheep receiving a single oral dose of meloxicam as 15 mg tablets have been described with a 72% bioavailability and serum concentrations remaining above 1 µg/mL for over 12 h [6]. Injectable meloxicam formulations have been associated with decreases in pain and inflammation in sheep [1,2] although no median effective (ED50) dose has been established for sheep. Plasma concentrations after repeated oral meloxicam dosing of sheep are lacking.

Meloxicam is not currently approved by the Food and Drug Administration for use in sheep in the US, therefore it is necessary to follow the guidelines set forth by the Animal Medicinal Drug Use Clarification Act (AMDUCA) including calculating conservative withdrawal interval (WDI) recommendations based on scientific data [7]. Tissue depletion studies are necessary to create a robust, scientifically based WDI following extra-label use of any medications, however published tissue depletion studies are limited compared to live animal pharmacokinetic studies that rely mostly on serum or plasma concentration data. Current estimated pharmacokinetic parameters for sheep administered meloxicam were derived from plasma concentrations and are limited to a single oral, subcutaneous (SC) or intramuscular (IM) dose or multiple SC doses [1,2,6,8,9]. Single oral dose data has limited clinical application since many conditions requiring treatment with anti-inflammatory pain medication do not resolve in a single day, and multiday therapy is commonly warranted and prescribed by veterinarians. In addition, requests for WDI information following extra label drug use in small ruminants has increased, as demonstrated by greater numbers of submissions to the Food Animal Residue Avoidance and Depletion program for sheep and goats over the past decade [10]. To the authors’ knowledge, there are no published pharmacokinetic parameters estimated from meloxicam concentration data associated with the administration of multiple oral doses, nor are there tissue depletion data associated with the administration of meloxicam to sheep.

During the initial study investigation, plasma meloxicam concentrations obtained immediately prior to slaughter in the tissue depletion study were increased compared to previous time points obtained for those animals during the survival portion of the multidose plasma pharmacokinetic (PK) study. It is unclear how physiologic changes surrounding the time of slaughter may influence drug concentrations. The phenomenon of post-mortem drug redistribution (PDR) has been reported in the human literature, and refers to changes that occur in circulating drug concentrations after death [11,12]. Preliminary data and concepts derived from human literature on PDR suggest there could be an effect of the slaughter process on terminal plasma sample concentrations. This phenomenon has not yet been explored in livestock.

Objective: The objectives of this study were to determine plasma pharmacokinetic parameters and obtain tissue residue depletion data for sheep administered oral meloxicam for ten daily oral doses of 1.0 mg/kg. These data were used to establish and compare WDI recommendations based on both terminal tissue residue depletion, and live animal plasma studies. An additional study objective was to investigate the association of the events surrounding the slaughter process with plasma sample meloxicam concentrations.

## 2. Materials and Methods

### 2.1. Animals

**Figure 1:**
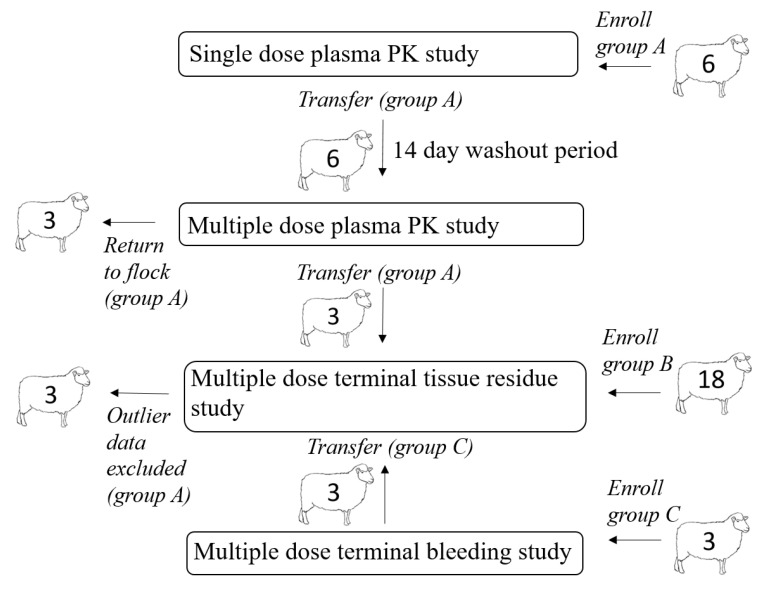
Relationship between animal use in each portion of the study.

A total of 27 yearling sheep (16 non-pregnant, non-lactating ewes and 11 wethers) determined to be healthy based on physical examination were enrolled in this study. All sheep were housed at the University of California, Davis Animal Science Sheep Facility and fed alfalfa hay ad libitum with free access to fresh drinking water. Free access to forage and water was provided to maintain consistent rumen fill and motility throughout the study. The relationship between animal enrollments in different portions of the experimental design is illustrated in Figure 1. After completion of the live animal sampling protocols, remaining sheep were either returned to the facility flock or sent through slaughter at a USDA accredited slaughter facility for terminal tissue residue depletion sampling, as determined by the study protocol. All procedures were approved by the Institutional Animal Care and Use Committee of the University of California at Davis (IACUC approved protocol #21274).

Numerals indicate animal numbers. A total of six sheep were included in the single dose live animal plasma PK study (group A); after the washout period these sheep were included in the multiple dose live animal plasma PK study. Three sheep from the multiple dose live animal PK study were slaughtered at the 192-h time point and samples were harvested for the tissue residue study. These animals were ultimately excluded from the data analysis. An additional 18 sheep (group B) were enrolled in the tissue residue depletion study. An additional 3 sheep (group C) were enrolled in a multiple dose terminal bleeding study; these sheep (group C) were included in terminal tissue residue study at slaughter.

A total of six sheep (group A) were included in the single dose live animal plasma PK study, and after a 14-day washout period, the same sheep (group A) were included in the multiple dose live animal plasma PK study (Figure 1). Three sheep from the multiple dose live animal PK study (group A) were eligible for slaughter and were slaughtered 192 h after the last dose time point and samples were harvested for the tissue residue study (Figure 1). These animals were ultimately excluded from all data analysis due to their increased plasma drug concentrations (which had previously been below the limit of detection (LOD)) immediately prior to slaughter; this drug behavior was considered abnormal and thus data from these animals was labeled as outlier. Eighteen additional sheep (group B) were enrolled in the tissue residue depletion study and slaughtered at pre-determined time points (Figure 1). An additional three sheep (group C) were enrolled in a multiple dose terminal bleeding study to evaluate the potential effects of procedures related to slaughter on plasma drug concentrations (Figure 1); the tissues from group C sheep were included in the tissue residue analysis at the 48 h post dosing time point. Concentration versus time data for group C sheep were not included in determining PK parameters for live animals treated multiple times.

### 2.2. Experimental Study Design

Two live animal PK studies (single and multi-dose) and one tissue drug depletion study (multi-dose) were initially performed. The live animal PK studies were conducted to obtain plasma concentration versus time data to estimate PK parameters for sheep administered single and multiple oral doses of oral generic meloxicam tablets (meloxicam 15 mg tablets, Cipla USA Inc, Warren, NJ; Meloxicam 7.5 mg tablets Zydus pharmaceutical, Pennington, NJ, USA. A tissue drug residue depletion study was also conducted to obtain tissue concentration versus time data for estimating a WDI. To investigate discordant meloxicam concentrations between plasma samples taken during live animal PK studies and immediately before and during slaughter, three additional animals were studied. These three animals were enrolled in a smaller study to investigate the association between the events surrounding the slaughter process and meloxicam plasma concentrations.

The study dose and administration route for all animals was 1 mg/kg meloxicam orally, via oral drench syringe (Stainless steel drench tip, Neogen, Lexington KY and BD 30 cc single use non-sterile syringes, Becton Dickinson & Co, Franklin Lakes, NJ, USA), once daily as single or repeated doses. Individual animal calculated doses were rounded to the nearest whole tablet (either 15 mg or 7.5 mg tablets) to establish the administered dose. Tablets were placed in the dosing syringe and dissolved using 20 mL of water for initial drug administration. Immediately following oral administration of the dissolved tablets, another 20 mL of water was drawn into the same dosing syringe and administered orally to flush the remaining drug from the syringe. Syringes were visually examined post dosing to ensure all of the drug had been administered. The study dose of 1 mg/kg was determined based on extra-label drug use withdrawal requests submitted to US FARAD. Published dose recommendations for ruminant species range from 0.35–1 mg/kg body weight for single oral doses without significant adverse effects noted [6,13,14,15,16,17,18,19,20,21,22,23,24]. Sheep were weighed at the start of each study and the meloxicam dose calculated and then weighed again after the last dose to confirm dose accuracy.

### 2.3. Live Animal Pharmacokinetic Studies

#### 2.3.1. Single Dose PK Study

Animal subjects consisted of six healthy adult sheep; three non-lactating, non-pregnant ewes and three wethers, with body weights ranging from 59 to 80 kg (mean, 72 kg) during the single dose study. Meloxicam was administered at 1 mg/kg meloxicam via oral drench syringe once. The dose was rounded to the nearest whole tablet, with an actual mean dose of 1.02 mg/kg (+0.02). Blood samples were obtained for analysis at 0.5, 1, 2, 4, 6, 8, 10, 12, 24, 48, 72 and 96 h after the single oral dose.

The same animals were used for both the single and multi-dose plasma PK portion of the study with a 14-d washout period between studies (Figure 1). A 14-d wash out period was deemed sufficient to allow complete drug depletion between studies based on existing PK data available for oral meloxicam treatment in sheep [6]. In addition, time zero plasma samples were obtained to ensure that no drug residues existed at the start of the multi-dose study.

#### 2.3.2. Multiple Dose PK Study

Animal subjects consisted of the same six healthy adult sheep used for the single dose study; three non-lactating, non-pregnant ewes and three wethers, with body weights ranging from 65 to 80 kg (mean 73 kg) during the multi-dose study. Meloxicam was administered to study sheep at 1 mg/kg meloxicam via oral drench syringe every 24 h for 10 doses. The dose was rounded to the nearest whole tablet, with an actual mean dose of 1.00 mg/kg (+0.04). Blood samples were obtained for analysis at 0.5, 1, 2, 4, 6, 8, 10, 12, 24, 48, 72, 96, 120, 144 and 192 h following the 10th (final) dose. The primary study purpose was to investigate tissue residue depletion after dosing regimen completion, thus sampling for drug concentration measurements prior to dosing completion was not performed. One sheep was not bled at 4 h post dosing due to fractious behavior. Three wethers from this group were enrolled in the tissue depletion study and were sacrificed at 192 h after the last dose. The final blood sample prior to slaughter was taken immediately prior to entering the slaughter floor (alley).

#### 2.3.3. Blood Collection

Blood samples were obtained via jugular venipuncture using needles and vacutainer 10.0 mL sodium heparin blood tubes. Tubes were placed on ice, centrifuged at 2730× *g* for 10 min at 21 °C, and plasma samples were manually harvested and transferred to storage tubes, which were then immediately frozen and stored at −70 °C until analysis.

#### 2.3.4. Tissue Residue Depletion Study

Animals consisted of 21 healthy adult sheep; 12 non-lactating, non-pregnant ewes and 9 wethers, with body weights ranging 55 to 79 kg (mean 67 kg). Three of the 9 wethers were previously enrolled in the multiple dose plasma PK study as described earlier (Figure 1). Meloxicam was administered at a dosage of 1 mg/kg meloxicam via oral drench syringe drench syringe every 24 h for 10 doses. The dose was rounded to the nearest whole tablet (either 15 or 7.5 mg), with an actual mean dose of 0.99 mg/kg (+0.04). The sheep were sacrificed in groups of three animals per time point, based on a minimum of three animals per time point needed for tissue residue depletion studies [25]. Sheep were slaughtered at 24, 48, 72, 96, 120, 144, 196 h after the tenth (last) dose. All study animals were fasted for 24 h prior to slaughter as standard procedure. Sheep were shipped to the slaughter facility the day of slaughter, with the exception of the sheep slaughtered at the 192 h time point, which were shipped to the slaughter facility the evening prior to slaughter. At time of slaughter, 8 mL of blood was obtained during terminal exsanguination. In addition, the entire liver, both kidneys and an approximately 200 g sample of leg muscle, loin muscle, body fat and renal fat was collected from each sheep and stored at −70 °C. Control sheep tissue for analysis was obtained from the campus meat laboratory and confirmed to be free of meloxicam.

#### 2.3.5. Terminal Bleeding Study

Following preliminary results showing a discrepancy between plasma sample meloxicam concentrations obtained during survival PK studies and meloxicam concentrations from plasma samples obtained immediately prior to slaughter in the tissue residue depletion study, three additional animals were added to the study to further examine these differences. Animals consisted of three healthy adult sheep; one non-lactating, non-pregnant ewe and two wethers, with body weights ranging 55 to 69 kg (mean 61 kg). Meloxicam was administered to study sheep at a dosage of 1 mg/kg meloxicam via oral drench syringe drench syringe every 24 h for 10 doses. The dose was rounded to the nearest whole tablet (15 mg tabs), with an actual mean dose of 1.05 mg/kg (+0.03). To enable repeated blood sampling immediately prior to and during terminal exsanguination, 16 g IV catheters (Arrow^®^ Central Venous Catheterization Kit, Arrow International, Reading PA, USA) were aseptically placed in a jugular vein of each animal at the beginning of the study. Blood collection was performed by aspirating 4 mL waste in a syringe, followed by a 10 mL blood sample which was immediately transferred to a vacutainer 10.0 ml sodium heparin blood tube, and the catheter was flushed with 6 mL heparinized saline. Blood samples were obtained for analysis at 0.5, 1, 2, 4, 6, 8, 10, 12, 24, and 48 h after the 10th and final dose. These animals were slaughtered at 48 h post final dosing, to ensure quantifiable drug concentrations. Additional blood collection time points surrounding the slaughter period were added to investigate the effect of the slaughter process on plasma sample drug concentrations in these additional animals. Blood samples were obtained from the IV catheters the day of slaughter a total of five times including: in their home environment prior to transport (barn), in the alley immediately prior to entering the slaughter floor (alley; similar to the previous animals included in both the multiple dose plasma PK and tissue depletion studies), immediately following captive bolt application (captive bolt), during terminal exsanguination samples were collected from catheters as the cervical vascular including both jugular and carotid vessels were transected rostral to the catheters (exsanguination), and a free catch sample was obtained at the very end of terminal exsanguination from the cervical transection site (free catch). All study animals were fasted for 24 h prior to slaughter as standard procedure. Sheep were shipped to the slaughter facility on the day of slaughter.

### 2.4. Chemicals and Materials

Meloxicam standard was obtained from European Pharmacopoeia Reference Standard (EDQM, Strasbourg, France). Piroxicam (Alfa Aesar, Ward Hill, MA, USA) was used as the internal standard. High performance liquid chromatography (HPLC)-grade methanol and acetonitrile, dimethyl sulfoxide, potassium phosphate monobasic, phosphoric acid and sodium sulfate were obtained from Fisher Scientific (Fair Lawn, NJ, USA). Purified water was obtained with a Nanopure water system (Barnstead, Dubuque, IA, USA).

### 2.5. Analytical Methods

Plasma and tissue concentrations of meloxicam were measured using an HPLC method with an ultraviolet detector. The HPLC system consisted of an Alliance 2695 separations module and 2487 dual wavelength absorbance detector and separation was achieved on a Nova-Pak C18, 4-µm, 300 × 3.9 mm column (Waters, Milford, MA, USA). Chromatographic conditions and preparation of standards and quality control samples was adapted from Kimble et al., 2012 [26]. Tissue sample clean-up was adapted from Sorensen and Hansen, 1998 [27]. The column temperature was maintained at 30 °C and the samples were kept at 10 °C. The isocratic mobile phase was 50:50 50 mM potassium phosphate buffer (pH 2.15) and acetonitrile set at a flow rate of 0.8 mL/min. Injection volume was 50 µL. Peaks were detected at a wavelength of 355 nm and the total run time was 10 min.

### 2.6. Preparation of Standards and Quality Control Samples

A primary stock solution of meloxicam (1.0 mg/mL) was prepared in dimethyl sulfoxide. This was diluted to a secondary stock solution (0.1 mg/mL) in 50% methanol (1:1 methanol to water) weekly. The secondary stock solution was used to create a series of working standard solutions (40 to 20,000 ng/mL), also in 50% methanol, and were prepared fresh for each analysis. A primary stock solution of piroxicam (1.0 mg/mL) in dimethyl sulfoxide and a secondary stock solution (0.1 mg/mL) in 50% methanol were similarly prepared. A 500 ng/mL working solution in 50% methanol was diluted from the secondary stock solution. Equal volumes of the meloxicam working solutions and the internal standard working solution were mixed for the standard curve (20 to 2500 ng/mL or 16 to 400 ng/mL, plasma or tissue). Three different concentrations of quality control samples (20, 100, and 400 ng/g or ng/mL) were prepared in control matrix with each analysis along with a matrix blank. Control matrices were collected from liver, kidney, muscle and fat from a non-medicated sheep at the time of slaughter. Control plasma was harvested from venous blood collected prior to slaughter.

### 2.7. Sample Preparation

Tissues were first processed with a Cuisinart food processor (Conair Corp., Stamford, CT, USA). Duplicate 1 g aliquots were weighed into centrifuge tubes and spiked with 200 µL of the working internal standard solution. Samples were then homogenized in 20 mL of acetonitrile with a Polytron Tissue Homogenizer (Kinematica, Bohemia, NY, USA). 10 mL of hexane was added, and the samples were shaken on a platform shaker for 10 min at 250 rpm. After centrifugation at 1200× *g* for 10 min, the hexane was removed to waste, and the acetonitrile was transferred to a 25 mL volumetric flask. Samples were brought to volume, added to 15 g of anhydrous sodium sulfate, shaken for one minute and centrifuged again at 1200× *g* for 10 min. A 12.5 mL aliquot of the extractant was evaporated to dryness at 60 °C with a gentle stream of nitrogen, reconstituted with 200 µL of 50% methanol and centrifuged at 12,000× *g* for 5 min before analysis on the HPLC system.

Plasma samples (250 µL) were spiked with 50 µL of the working internal standard solution prior to the addition of 3.5 mL of acetonitrile. After vortex mixing, the samples were centrifuged at 1200× *g* for 10 min. The extractant was transferred to a new tube and evaporated to dryness at 60 °C with a gentle stream of nitrogen, reconstituted with 100 µL of 50% methanol and centrifuged at 12,000× *g* for 5 min before analysis on the HPLC system.

### 2.8. Method Validation

Representative calibration plots and chromatograms for analysis of meloxicam in sheep plasma and tissues (liver, kidney, muscle and fat) are available in the Supplementary Materials (Figures S1–S5). Plasma and kidney were validated according to the FDA Bioanalytical Method Validation Guidance for Industry [28]. Intra–day precision was calculated on a single day using five replicates at each concentration. Inter-day precision was calculated using five replicates at each concentration over three consecutive days. Calibration curves were using the ratio of meloxicam to the internal standard peak areas and had a 1/X2 weighting. The average R squared was 0.9965 for the plasma assay and 0.9962 for the tissue assays. Limit of detection (LOD) was measured by adding three times the standard deviation of baseline measurements to the average baseline measurement using the blank QCs analyzed with each sample set. The lower limit of quantification (LLOQ) was measured by taking five times the baseline measurement, as per the FDA Guidance for Industry. The three quality control levels described above under, “Preparation of standards and quality control samples” were also used to measure precision and accuracy of the method concurrent with sample analysis. Intra assay precision during sample analysis was 3.2%, 2.5%, 3.0%, 2.6% and 4.3% for plasma, muscle, fat, kidney and liver, respectively, using relative standard deviation. Inter assay precision was 6.3%, 4.8%, 7.0%, 5.3% and 6.1% for plasma, muscle, fat, kidney and liver, respectively. Average accuracy was 101.9%, 101.4%, 95.9%, 99.2% and 99.7% for plasma, muscle, fat, kidney and liver respectively. Expanded precision and accuracy data is available as a supplementary file. Individual values for precision, as measured by relative standard deviation, and accuracy data obtained during quality control analysis is provided in the Supplementary Materials (Table S1). Method validation values for precision and accuracy of plasma and kidney are provided in the Supplementary Materials (Table S2).

### 2.9. Data Analysis

Meloxicam plasma concentration versus time data from the single and multiple dose live animal PK studies were analyzed using commercial modeling software (Phoenix 8.1, Certara, Princeton, NJ, USA). The C_max_ and T_max_ were observed from the time vs. concentration data for each animal in each study. The T_1/2_ was estimated using the equation T_1/2_ = ln(2)/k_el_. The λz and area under the plasma concentration-time curve (AUC), from time 0 to infinity and the percent extrapolated from the area under the plasma concentration-time curve from time 0 to the last quantifiable measurement, were estimated using non-compartmental methods performed by the Phoenix PK/PD modeling software (El Segundo, CA, USA). The AUC was analyzed using the linear up and log down approach. Terminal elimination pharmacokinetic parameters were estimated based on best fit data. The apparent volume of distribution during the terminal elimination phase (Vz/F) was calculated based on the terminal phase and used the equation Vz/F_(obs)_ = dose/[λz(AUC_inf_)]. The apparent total clearance of drug from plasma (CL/F_(obs)_) was calculated using the equation CL/F = dose/AUC_inf_. The area under the first moment of the plasma concentration-time curve from time zero to infinity (AUMC_inf(obs)_) was calculated using the equation AUMC_inf(obs)_ = AUMC_last_ + [(t_last_ × C_last_)/λz] + (C_last_/λz^2^). The mean residence time, based on concentration-time curve extrapolated to infinity (MRT_inf(obs)_) was calculated for a non-infusion model dosing using the equation MRT_inf_ = AUMC_inf_/AUC_inf_. Parameters were not normally distributed and were thus reported as median (range). All PK parameters were compared between the single and multiple dose plasma PK studies using the Wilcoxon sign rank test to assess for differences between PK parameters after single vs. multiple dosing; significance was set at *p* ≤ 0.05. The mean Cmax after the 10th dose of meloxicam was compared to the previously reported, potentially therapeutic, concentration of 1 µg/mL via a one sample t-test to assess if the dosing regimen reported herein was consistent with potentially therapeutic dosing.

Tissue concentration versus time data, collected from slaughter at predetermined times, were analyzed to create withdrawal intervals. Since there is currently no tolerance for meloxicam in livestock matrices in the United States the LOD for each tissue was considered the tolerance for each calculation. Following FDA regulatory guidance, withdrawal intervals were calculated using the tolerance limit method and all points less than LOD were excluded as previously described and developed by the FDA [25]. Analysis was conducted in R (R Core Team, 2104, Vienna, Austria) using the “ResChem” package [25]. Linear modeling of the logarithmic concentration versus time were run for each permutation of inclusion of time points. Points violating log linearity were removed; the model with the lowest *p*-value was considered the best linear model. An upper 99% percentile tolerance band with a 95% confidence of the logarithmic concentrations was included in the linear model. The log_e_ transformed tolerance was added in the plot to calculate where this log_e_ transformed tolerance crosses the upper 99% percentile tolerance band. Thus, the withdrawal time is extrapolated from the intersection of these two lines. Estimated WDIs were rounded up to the nearest whole day. For comparison, the European Medicines Agency (EMA), Approach Towards Harmonization of Withdrawal Periods Calculation [29] was also used to estimate a WDI. Values less than LOD were excluded. Values less than LLOQ, but greater than the LOD were replaced with LLOQ/2 per established guidelines [29]. The maximum residue limit established in the EU for liver and kidney of goats (65 µg/kg) was used for the EMA calculations [30]. All estimated WDIs were rounded up to the nearest whole day. For additional comparison, WDI estimates were created using plasma sample meloxicam concentrations by two additional methods. Plasma concentrations of meloxicam from samples obtained at terminal exsanguination at slaughter between 1 and 6 d following the last day of treatment were analyzed using the FDA tolerance method described above for estimation of a WDI. Additionally, a WDI for plasma samples taken from live animals during the multiple dose PK study was estimated by multiplication of the median terminal elimination half-life (T_½_) by ten, when 99.9% of the drug would be expected to be eliminated from the body.

To further analyze the association of events surrounding the slaughter process and plasma meloxicam concentrations, two types of additional statistical comparisons were made. Initially, meloxicam concentrations between plasma samples obtained at the time of terminal exsanguination during slaughter and plasma obtained from survival bleeding time points during the plasma PK study at matched time points were compared using the ANOVA and Welch’s ANOVA, depending on equality of data point variance. Additionally, meloxicam concentrations in plasma samples were compared between the five sequential sampling time points surrounding the slaughter process (barn, alley, captive bolt, exsanguination, free catch) obtained from the additional three animals added to the study using a Welch’s ANOVA.

## 3. Results

**Figure 2:**
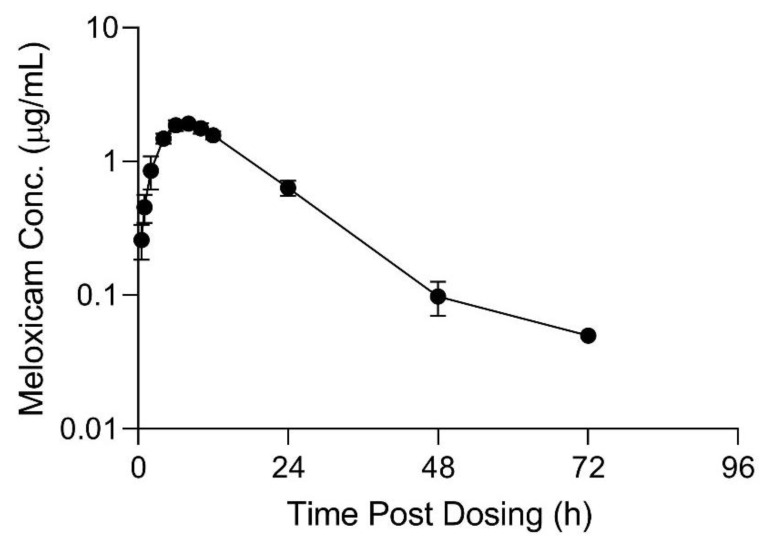
Mean plasma meloxicam concentrations (+SD) sampled over time (0–96 h) for six sheep administered a single oral 1 mg/kg dose of meloxicam tablets dissolved in water. Values below the lower limit of quantitation and limit of detection are excluded.

**Figure 3:**
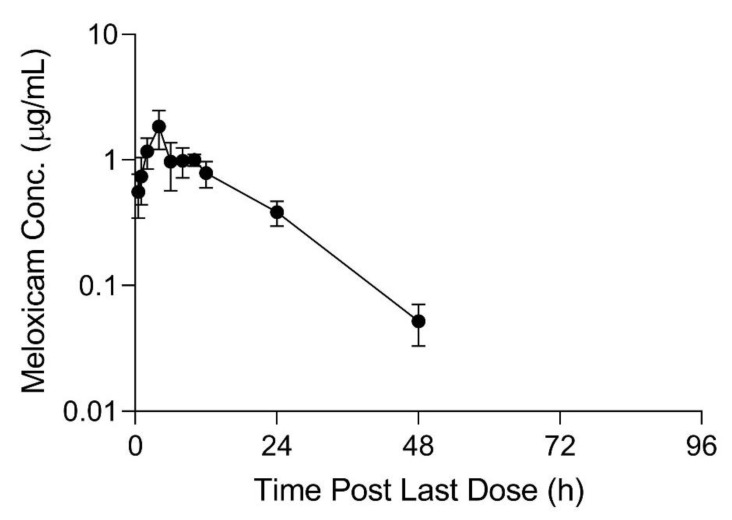
Mean plasma meloxicam concentrations (+SD) sampled over time (0–96 h) for six sheep administered 10 daily oral 1 mg/kg doses of meloxicam tablets dissolved in water. Values below the lower limit of quantitation and limit of detection are excluded.

**Figure 4:**
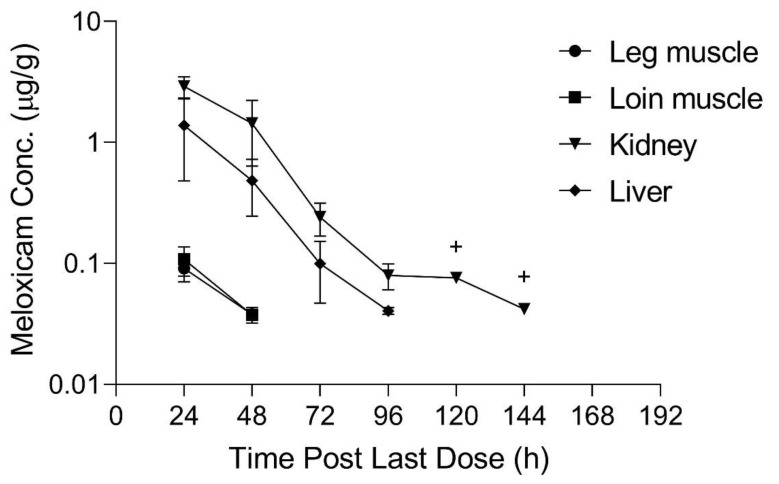
Mean tissue meloxicam concentrations (+SD) sampled over time (0–192 h) for sheep after administration of 10 daily oral 1 mg/kg doses of meloxicam dissolved in water. N = 3 sheep per time point at 24, 72, 96, 120, 144 h and n = 6 at 48 h, 21 total sheep. Values below the lower limit of quantitation or limit of detection are excluded. Single data point indicated by +, therefore no SD bars provided.

The limit of detection (LOD) and lower limit of quantification (LLOQ) for all sample types is displayed in Table 1. Pharmacokinetic parameters estimated from plasma sample concentrations versus time from the live animal pharmacokinetic studies are displayed in Table 2 and Table 3. Meloxicam concentrations over time in plasma and tissue samples after completion of dosing are displayed in Figure 2, Figure 3 and Figure 4. Plasma meloxicam concentrations had more variability following multi-dose therapy compared to single dose therapy, as observed by the variation in the time vs. concentration curves between Figure 2 and Figure 3. The parameters, C_max(obs)_, λz, and T_½_ did not demonstrate significant differences between the single and multiple dose plasma PK studies. The PK parameters Vz/F_(obs)_, CL/F_(obs)_, AUMC_inf_, MRT_inf_, AUC_0-inf_, AUC_last_, and T_max_ were different between the single and multiple dose plasma PK studies (*p* = 0.03). There is some evidence for decreased overall drug exposure following multiple doses as demonstrated by the decrease in AUC_0-inf_ for the multi-dose study compared to the single dose study. The Vz/F_(obs)_, CL/F_(obs)_ were greater following multiple dosing compared to following a single dose. The AUMC_inf(obs)_ and MRT_inf(obs)_, and T_max_ were greater following a single dose compared to multiple dosing. The mean observed plasma C_max(obs)_ 24 h after dosing of meloxicam was greater than 1 µg/mL in both the single and multi-dose plasma PK studies (*p* = 0.0006). Five of 12 animals had plasma sample concentrations at or above one µg/mL at 24 h after the final dose. The highest tissue concentrations after 10 daily doses were observed in the kidney at 24 h after the final dose.

The estimated slaughter WDI recommendation after 10 daily 1 mg/kg oral doses of meloxicam in sheep is 219 h (10 d), using the FDA tolerance method for kidney meloxicam concentrations. Kidney, plasma and liver samples had values sufficient for WDI calculation using the FDA regulatory method (219, 198 and 182 h, respectively); the longest WDI estimate was for kidney. Due to insufficient data points, WDIs could not be established for other tissues. The three animals slaughtered at 192 h after the 10th oral dose of meloxicam were considered outliers, due to the rise in meloxicam plasma concentrations at the time of slaughter compared to previous time points and were not included in WDI calculation analysis. The additional three animals added to the study to investigate the effect of slaughter on plasma meloxicam concentration were also not included in the WDI calculations. A total of 18 animals at 6 time points were thus included in the final WDI analyses (Figure 1).

For comparison, using the EMA method for tissue residue depletion, the WDI recommendation would be 144 h (6 d) based on kidney sample data. Kidney and liver samples had sufficient data points for estimating WDIs using the EMA method (144 and 117 h, respectively); the longest WDI estimate was for kidney samples.

For further comparison, the estimated WDIs based on plasma samples taken from terminal exsanguination at slaughter between 1 and 6 d following the last day of treatment, using the FDA tolerance method for these plasma meloxicam concentrations was 198 h (9 d). The WDI for plasma samples taken from live animals during the multiple dose PK study, using ten times the elimination half-life (T_½_) was 92 h (4 d).

**Figure 5:**
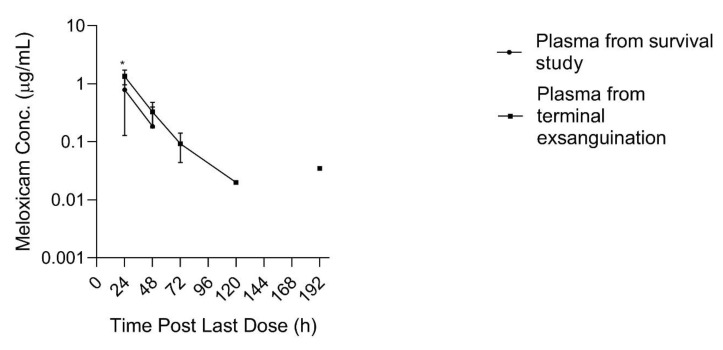
Plasma sample meloxicam concentrations (+SD) after 10 daily oral 1 mg/kg doses obtained either via live animal phlebotomy in a survival PK study or at time of terminal exsanguination and tissue collection in a tissue depletion study. Live animal phlebotomy n = 6; terminal exsanguination n = 3 per time point, 21 sheep total. Data points below the LLOQ excluded. No meloxicam was detected in any plasma samples in the live animal study at 120,144 and 192 h. Significant difference between groups at 24 h indicated by an * (*p* = 0.045).

At 192 h after the 10th dose, two out of three plasma samples obtained immediately prior to entering the slaughter floor contained meloxicam concentrations greater than the LLOQ (0.035 and 0.035 µg/mL) which appeared greater than concentrations obtained at the previous time point (168 h) for the same animals which were all below the LLOQ, and likewise appeared greater than the plasma concentrations obtained from the other three animals sampled at 192 h post dosing during the multi-dose plasma PK survival study, which were all likewise below the LLOQ. Plasma meloxicam concentrations, at 24 h post dosing, obtained from terminal blood collection at slaughter during the tissue depletion study were greater than time matched plasma concentrations obtained from the animals in the multi-dose PK survival study, (*p* = 0.045) as displayed in Figure 5. Plasma drug concentrations obtained from three additional sheep, sampled in the follow-up study throughout different stages of the transport, stunning and exsanguination process 48 h after final dosing, demonstrated no differences in plasma meloxicam concentrations between the five time points sampled surrounding the slaughter process (barn, alley, captive bolt, exsanguination, free catch).

## 4. Discussion

This manuscript reports estimated PK parameters for live animal studies after multiple daily oral doses of meloxicam administered to sheep. A dose of 1 mg/kg orally for 10 d appears to provide potentially therapeutic plasma concentrations above 1µg/mL, however the duration above this threshold was not greater than 24 h for all animals. The concentration vs. time curve following multiple daily oral doses has greater variability than after a single dose which was reflected in the estimated PK parameters. The median AUC_0-inf_ from the multiple dose study was less than after the single dose study, the median C_max_ was likewise slightly less (however C_max_ ranges overlapped between studies), however the T_1/2_ remained similar between the two studies. This suggests that enzymatic auto-induction did not occur after multiple dose administration. This basis for the AUC and C_max_ differences is unknown but may have been due to individual animal differences between the single dose and multi-dose trials. For both trials, the same formulations of meloxicam tablets were used, and oral dosing was provided with the same ad-lib feeding schedule.

Using the FDA tissue tolerance method, a 10 d WDI estimate for slaughter in sheep following multiple daily oral 1 mg/kg meloxicam dosing is recommended based on kidney tissue concentrations. The use of the FDA method described herein is recommended for use in the United States because there is no tolerance limit for meloxicam in the edible tissues of sheep; this method is most conservative and least likely to result in detectable or violative tissue residues. In comparison, the EMA method calculates a WDI recommendation of 144 h (6 d) based kidney sample data; the EMA method converts all data points below LOQ to half of the LOQ thus impacting the WDI estimate.

In the absence of tissue depletion studies, an estimated WDI can be calculated from plasma PK studies but is less than optimal if plasma to tissue ratios are unknown. Plasma sample WDI estimates were 9 d and 4 d using terminally obtained plasma and plasma from the live animal PK study, respectively; the live animal PK study underestimates the WDI compared to the FDA method using tissue samples in this study. It is thus imperative to incorporate tissue residue depletion when possible, to make the most informed WDI’s possible. Additionally, the plasma samples taken from terminal exsanguination during slaughter (WDI estimate of 9 d) seems to more closely reflect the tissue depletion (WDI estimate of 10 d based on kidney tissue), whereas the 4 d WDI estimate using samples taken during the live animal study is not as conservative. This finding is interesting because the live animal plasma PK study should provide more reliable data since all 6 animals were sampled throughout all time points, whereas in the non-survival tissue harvest study only three animals were enrolled per time point.

The spuriously high plasma sample concentrations of meloxicam at 192 h post 10 d of dosing, and the apparently greater drug concentrations found in plasma obtained during terminal exsanguination compared to plasma samples obtained during survival phlebotomy (Figure 5), prompted investigation into the association between events surrounding the slaughter process and plasma meloxicam concentrations. The terminal exsanguination study demonstrated no differences in plasma drug concentration between time points during the slaughter process (taken at 48 h post final dose). It is unclear why the terminal exsanguination study yielded conflicting results to the initial study data. It is possible that differences in housing and shipping may have influenced the results. Sheep from the 192 h slaughter time point were transported to the slaughter facility the day prior to slaughter and kept in an on-site holding pen until slaughter, while sheep in all other portions of the study, including the terminal exsanguination study, were shipped to the slaughter facility on the day of slaughter. The preliminary data observations, and inconsistency with the follow-up terminal exsanguination study, raise the question of whether physiologic changes surrounding the stress of housing changes, shipping, or slaughter procedures may influence plasma sample drug concentrations and warrants further exploration. A study in horses at slaughter found that horses have physiologic changes consistent with stimulation of the sympathetic nervous system (SNS) during stunning and exsanguination [31]. Serum total protein and albumin concentrations were significantly higher than periods leading up to stunning in that study. Meloxicam is metabolized in the liver and the proximity of the liver to major vessels may also facilitate a redistribution of meloxicam to the circulation during catecholamine response during stressful events, such as events surrounding slaughter.

A potential limitation of this study was the difference in animal management the 24 h immediately preceding slaughter and terminal sampling, which may be responsible for surprising terminal plasma sample increases in meloxicam concentration. Additionally, the small sample size used in the terminal bleeding study limited our ability to assess normality of the data and therefore the limitations of potentially non-normal distribution and repeated measures exist. Calculation of a WDI by FDA methods was limited by the lack of an existing tolerance limit for meloxicam in tissues of sheep. Calculation of a WDI by FDA methods without a tolerance limit requires the use of the LOD as the tolerance, and this method inherently prevents analysis of data points below the LOD due to the inherent lack of reliability of data points below the LOD. Additionally, the determination of tissue meloxicam concentrations was performed without a correction for the drug content in residual blood. Additional variation may have been introduced by the use of two different sizes of meloxicam tabs made from different drug manufacturers. Another potential limitation of this study is that trough concentration prior to dosing were not obtained to verify the 24 h dosing interval, however plasma and tissue concentrations were obtained following the 10th and final dose to observe drug elimination over time, as needed for WDI calculation. The observation of this information at 24 h after completion of dosing provides insight into the clinical utility of a 24 h dosing interval. Once daily dosing is the dosing interval most commonly used and most practical for the treatment of livestock; this regimen appears reasonable based on a mean plasma C_max_ > 1 µg/mL at 24 h, however it is not ideal for all animals since some animals fell below a presumed therapeutic concentration of 1 µg/mL by 24 h after dosing.

The PK parameters described in our study are compared to other pharmacokinetic reports using oral meloxicam in ruminants or pseudo ruminants in Table 4. Several plasma PK parameters including C_max_, λz, and T_½_ from the single oral dose study are similar to previous reports in this species [6]. The median T_max_ after oral meloxicam dosing in our study 8 h in the single dose study and 4 h in the multi-dose study; this is notably less than other reports at 19 h in sheep [6] and approximately 90 h in a multi-dose study in calves [32]. The relatively rapid increase in plasma sample meloxicam concentrations in our study is unlikely related to the route of administration because these studies likewise used tabs dissolved in water and administered the resulting solution by oral drench [32] or oro-ruminal tubing [6]. This relatively rapid increase may be related to differences in tablet formulation; the excipients in the 7.5 mg tabs used in this study are the same as that reported by Stock et al. [6], however the 15 mg tabs used contained different excipients than reported by either Stock et al. [6] or Coetzee et al. [32]; however, all of these are considered immediate release tablets. Differences in T_max_ may also be due to animal differences such as breed, species, feeding schedule or other differences in housing or management that may affect drug absorption between studies. The AUC_0-inf_ generally appears to be greater for cattle [14,15,19,20,21,32,33,34,35,36] than for the sheep in our study, which suggests greater drug exposure in cattle compared to sheep in this study.

## 5. Conclusions

This study provides PK data including novel tissue depletion data following the use of multiple doses of meloxicam orally in sheep. These data have been used to create a slaughter WDI of at least 10 d following multiple oral doses of meloxicam in sheep, which is critical for protecting the food supply after extra label drug use of multiple doses of meloxicam in sheep. This study also reports a novel finding that drug concentrations obtained from terminal blood collection at the time of slaughter may differ from time matched drug concentrations obtained from living animals, although this finding was not repeatable in our follow up investigation. Calculation of WDI recommendations may differ if based on terminally obtained plasma samples compared to samples obtained during a live animal plasma PK study.

## Figures and Tables

**Table 1:** Limit of detection (LOD) and lower limit of quantification (LLOQ) values. Data represents the high-performance liquid chromatography method used to quantify meloxicam concentrations in plasma and tissue samples from sheep following administration of single or multiple oral doses of 1 mg/kg meloxicam tablets dissolved in water.

Tissue Type	LOD	LLOQ
Plasma (µg/mL)	0.0049	0.020
Leg muscle (µg/g)	0.0073	0.025
Loin muscle (µg/g)	0.0073	0.025
Abdominal fat (µg/g)	0.0071	0.025
Carcass fat (µg/g)	0.0071	0.025
Kidney (µg/g)	0.0063	0.025
Liver (µg/g)	0.0071	0.025

**Table 2:** Median (range) for non-compartmental pharmacokinetic parameters estimated for sheep (n = 6) administered a single oral 1 mg/kg dose of meloxicam.

Parameter	Median (Range)
AUC_last_ (h·µg/mL)	36.66 (33.36–40.79)
AUC_0-inf_ (h·µg/mL)	38.15 (34.15–41.58)
AUC_0-inf_% extrapolated	2.8 (1.92–5.16)
C_max(obs)_ (µg/mL)	1.94 (1.73–2.12)
T_max(obs)_ (h)	8 (6–8)
λz (1/h)	0.073 (0.063–0.087)
T_½_ (h)	9.49 (8.01–11.05)
Vz/F (mL/kg)	357 (319–389)
CL/F (mL/h/kg)	26.2 (24.0–29.3)
AUMC_inf(obs)_ (h·h·µg/mL)	662 (541–750)
MRT_inf(obs)_ (h)	17.2 (15.9–18.0)

AUC_0-inf_ = Area under the plasma concentration-time curve from time 0 to infinity; C_max(obs)_ = Maximum observed plasma concentration; AUC_last_ = Area under the plasma concentration-time curve from time 0 to the last quantifiable measurement. T_max(obs)_ = Time at which the maximum plasma concentration was observed; λz = Elimination rate constant based on best fit data points; T_½_ = Elimination half-life based on best fit data points. Vz/F = apparent volume of distribution during the terminal elimination phase; CL/F = apparent total clearance of drug from plasma; AUMC_inf_ = area under the first moment of the plasma concentration-time curve from time zero to infinity; MRT_inf_ = mean residence time, based on concentration-time curve extrapolated to infinity.

**Table 3:** Median (range) non-compartmental pharmacokinetic parameters estimated for sheep (n = 6) administered 10 daily oral 1 mg/kg doses of meloxicam.

Parameter	Median (Range)
AUC_last_ (h·µg/mL)	23.1 (18.9–26.8)
AUC_0-inf_ (h·µg/mL)	23.9 (19.1–27.7)
AUC_0-inf_ % extrapolated	3.35 (1.15–3.72)
C_max(obs)_ (µg/mL)	1.65 (1.42–2.65)
T_max(obs)_ (h)	4 (2–8)
λz (1/h)	0.075 (0.066–0.10)
T_½_ (h)	9.20 (6.90–10.46)
Vz/F_(obs)_ (mL/kg)	546 (494–574)
CL/F_(obs)_ (mL/h/kg)	41.8 (36.0–52.3)
AUMC_inf(obs)_ (h·h·µg/mL)	383 (251–429)
MRT_inf(obs)_	15.2 (13.1–16.6)

AUC_0-inf_ = Area under the plasma concentration-time curve from time 0 to infinity; C_max(obs)_ = Maximum observed plasma concentration; AUC_last_ = Area under the plasma concentration-time curve from time 0 to the last quantifiable measurement. T_max(obs)_ = Time at which the maximum plasma concentration was observed; λz = Elimination rate constant based on best fit data points; T_½_ = Elimination half-life based on best fit data points. Vz/F_(obs)_ = apparent volume of distribution during the terminal elimination phase; CL/F_(obs)_ = apparent total clearance of drug from plasma; AUMC_inf(obs)_ = area under the first moment of the plasma concentration-time curve from time zero to infinity; MRT_inf(obs)_ = mean residence time, based on concentration-time curve extrapolated to infinity.

**Table 4:** Summary of published (as of December 2020) plasma PK parameters for meloxicam in ruminants and pseudo ruminants following oral administration of meloxicam. Data are reported as means except where indicated.

**Author**	**Current Study**	**Stock et al.** [6]	**Karademir et al.** [23]	**Ingvast-Larsson et al.** [22]	**Coetzee et al.** [32]	**Meléndez et al.** [33]	**Bublitz et al. ‡** [18]	**Kreuder et al.** [24]	**Glynn et al.‡** [20]	**Malreddy et al.** [19]	**Mosher et al.** [34]	**Mosher et al.** [34]
Publication Year		2013	2016	2010	2015	2019	2019	2012	2013	2012	2011	2011
Dosage (mg/kg)	1	1	0.5	0.5	0.5	1	0.5	1	1	1	0.6	0.6
Single or Multi-dose Administration	10 daily doses	Single	Single	Single	4 daily doses	Single	Single	Single	Single	Single	Single	Single
Species	Sheep	Sheep	Goat	Goat	Beef Calves	Beef Calves	Goats	Llama	Dairy Calves	Dairy Cows	Pre-ruminant calves	Ruminating calves
AUC_0-inf_ (µg·h/mL)	23.1	75.1	24.2	23.2	239	95.2	17.6	68.4	78.0	90.0	151	86.7
AUC% extrapolated	3.35	1.9	N/A	N/A	N/A	0.18	0.65	6.6	N/A	N/A	N/A	N/A
C_max_ (µg/mL)	1.65	1.72	0.71	0.74	4.52	2.33	0.7	1.314	1.90	2.89	2.2	1.95
T_max_ (h)	4	19.0	14.33	15	88.5	24	6.0	21.4	24	11.33	17	17.3
λz (1/h)	0.075	0.045	N/A	0.060	N/A	0.045	0.065	0.031	N/A	0.06	0.026	0.024
T _½_ (h)	9.20	15.4	10.7	11.8	25.7	15.6	10.7	22.7	16.7	14.6	40	29.9
**Author**	**Current Study**	**Coetzee et al.** [35]	**Coetzee et al.** [15]	**Wani et al.** [37]	**Allen et al.** [14]	**Allen et al.** [14]	**Gorden et al.** [21]	**Gorden et al.** [21]	**Shock et al.** [36]	**Warner et al.** [38]	**Warner et al.** [38]	
Publication Year		2009	2014	2014	2013	2013	2018	2018	2019	2020	2020	
Dosage (mg/kg)	1	1	0.5	0.35	1	1	1	1	1	1	1	
Single or Multi-dose Administration	10 daily doses	Single	Single	Single	Single	Single	Single	Single	Single	Single	Single	
Species	Sheep	Dairy Calves	Beef cattle	Goats	Dairy Calves, pre dehorning	Dairy Calves, post dehorning	Post-partum Dairy cattle	Mid lactation Dairy cattle	Post-partum Dairy cattle	Mid lactation Dairy cattle	Post-partum Dairy cattle	
AUC (0-inf) (µg·h/mL)	23.1	164.46	276.78	N/A	216.12	217.53	109.34	51.92	86.13	36.01	82.82	
AUC% extrapolated	3.35	16.71	N/A	N/A	N/A	N/A	N/A	N/A	N/A	0.6	0.47	
C max (µg/mL)	1.65	3.1	4.7	1.608	3.61	3.27	2.92	1.82	1.68	1.45	2.61	
T max (h)	4	11.64	89.6	8.28	13.95	15	17.6	11.6	13.33	10.48	16.75	
λz (1/h)	0.075	0.025	N/A	N/A	N/A	N/A	0.058	0.059	N/A	0.07	0.06	
T _½_ (h)	9.20	27.54	22.49	N/A	38.62	35.81	13.01	11.93	25.71	9.55	12.28	

‡ Original manuscript reported results as median.

## Data Availability

The data presented in this study are available on request from the corresponding author.

## Supplementary Materials

The following are available online at https://www.mdpi.com/article/10.3390/ani11102797/s1, Supplemental Figures: Representative calibration plots and chromatograms for analysis of meloxicam in sheep plasma and tissues (liver, kidney, muscle and fat). Table S1: Individual values for precision (relative standard deviation) and accuracy data obtained during quality control analysis for determination of meloxicam in plasma, muscle, fat, kidney and liver of sheep. Table S2: Individual values for precision (relative standard deviation) and accuracy data obtained during method validation analysis for determination of meloxicam in plasma and kidney of sheep.
