# Supplementary material for: Pharmacokinetic Parameters and Tissue Withdrawal Intervals for Sheep Administered Multiple Oral Doses of Meloxicam
Source: Animals (Basel). 2021 Sep 25;11(10):2797. doi: 10.3390/ani11102797 (PMC8532701; doi:10.3390/ani11102797)
Supplement: Supplementary file 1 [file animals-11-02797-s001.zip › animals-1259115-supplementary.pdf]

animals

MDPI

Supplemental Information

Table S1. Individual values for precision (relative standard deviation) and accuracy data obtained during quality control analysis for determination of meloxicam in plasma, muscle, fat, kidney and liver of sheep.

Quality control drug concentration	0.02 µg/mL or µg/g			0.1 µg/mL or µg/g			0.4 µg/mL or µg/g		
	Intra- assay variation (RSD) average (range) (%)	Inter-assay variation (RSD) (%)	Accuracy average (range) (%)	Intra- assay variation (RSD) average (range) (%)	Inter-assay variation (RSD) (%)	Accuracy average (range) (%)	Intra- assay variation (RSD) average (range) (%)	Inter-assay variation (RSD) (%)	Accuracy average (range) (%)
**Plasma**	3.9 (0.1–11.7)	6.5	101.5 (95.1–109.3)	2.6 (0.4–5.4)	5.9	99.6 (91.0–110.9)	3.3 (0–10.4)	6.4	101.5 (95.6–116.9)
**Muscle**	3.8 (0.8–8.9)	5.1	101.5 (96.0–106.5)	2.5 (0–10.7)	4.6	103.7 (98.6–108.3)	1.6 (0.4–2.9)	4.4	98.4 (95.1–106.6)
**Fat**	2.6 (0.3–5.9)	6.4	96.9 (92.9–102.2)	4.4 (1.7–10.2)	13.4	91.0 (67.1–100.9)	2.6 (0.1–7.0)	9.2	93.5 (75.5–103.4)
**Kidney**	3.9 (2.0–7.0)	6.3	102.2 (92.5–106.2)	3.0 (0.6–6.5)	6.6	97.4 (92.3–108.9)	1.8 (1.2–2.1)	5.1	98.7 (93.1–105.0)
**Liver**	6.1 (1.8–11.9)	6.5	100.3 (94.7–106.4)	2.8 (1.3–4.4)	3.1	96.3 (92.7–99.0)	1.7 (0.6–2.7)	7.3	100.2 (92.0–105.1)

Quality control was assessed with three standard dilutions of blank matrix (plasma, muscle, fat, kidney or liver) spiked with meloxicam; 0.02, 0.1 and 0.4 µg/mL or µg/g. The unit of measure were µg/mL for plasma and µg/g for muscle, fat, kidney and liver. Variation was calculated using the relative standard deviation (RSD), which was calculated using the equation RSD = standard deviation / average result of concentration testing X 100. Accuracy was calculated using the equation: accuracy = achieved concentration result/expected concentration result × 100. Duplicate samples at each QC concentration (0.02, 0.1 and 0.4 µg/mL) were run with each sample set. A total of 16 sets of plasma, 10 sets of muscle, 10 sets of fat, 6 sets of liver and 5 sets of kidney were run.

Table S2. Individual values for precision (relative standard deviation) and accuracy data obtained during method validation analysis for determination of meloxicam in plasma and kidney of sheep.

Plasma				Kidney			
Quality control drug concentration (µg/mL)	Intra- assay variation (RSD) average (range) (%)	Inter-assay variation (RSD) (%)	Accuracy average (range) (%)	Quality control drug concentration (µg/g)	Intra- assay variation (RSD) average (range) (%)	Inter-assay variation (RSD) (%)	Accuracy average (range) (%)
0.02	3.3 (1.8–4.3)	3.4	104.9 (103.7–106.5)	0.025	3.6 (2.9–4.2)	4.3	102.0 (99.0–105.3)
0.06	3.5 (1.0–6.3)	4.2	104.2 (101.9–106.2)	0.075	2.9 (2.2–4.4)	2.9	102.1 (102.0–102.1)
0.60	4.4 (3.8–5.3)	4.6	99.4 (96.6–101.0)	0.225	2.6 (1.4–3.5)	4.0	100.6 (96.6–103.5)
6.00	2.7 (2.0–3.1)	4.3	98.5 (95.3–103.1)	0.400	2.6 (1.9–3.0)	3.8	101.7 (97.7–103.7)

Quality control was assessed with four standard dilutions of blank matrix (plasma or kidney) spiked with meloxicam; 0.02, 0.06 0.60 and 6.00 µg/mL or 0.025, 0.075, 0.225 or 0.400 µg/g. The unit of measure were µg/mL for plasma and µg/g for kidney. Variation was calculated using the relative standard deviation (RSD), which was calculated using the equation RSD = standard deviation / average result of concentration testing X 100. Accuracy was calculated using the equation: accuracy = achieved concentration result/expected concentration result X 100. Five samples at each QC concentration were run with each sample set for a total of three sample sets.

Supplemental Figures. Representative calibration plots and chromatograms for analysis of meloxicam in sheep plasma and tissues (liver, kidney, muscle and fat)

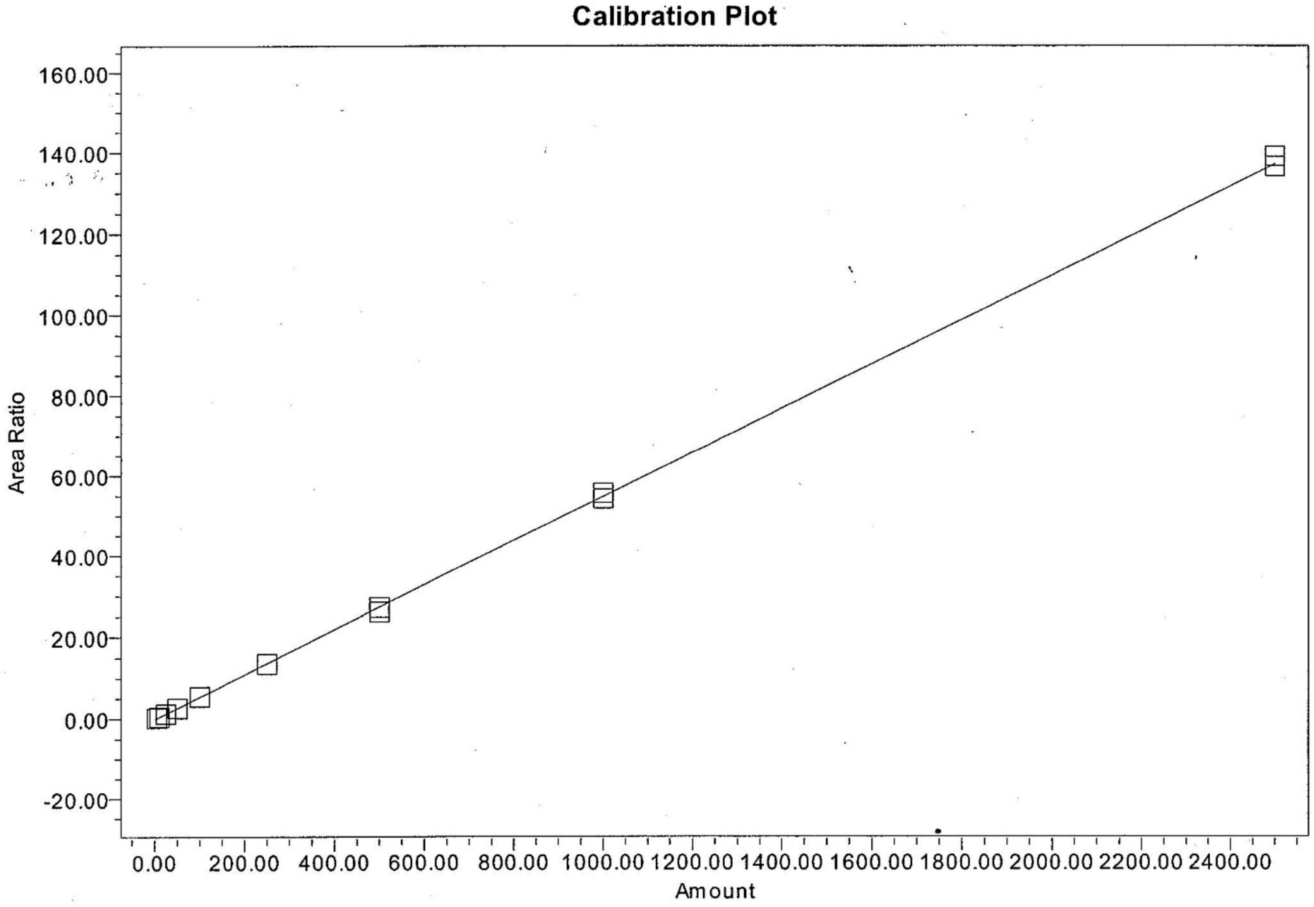

(a)

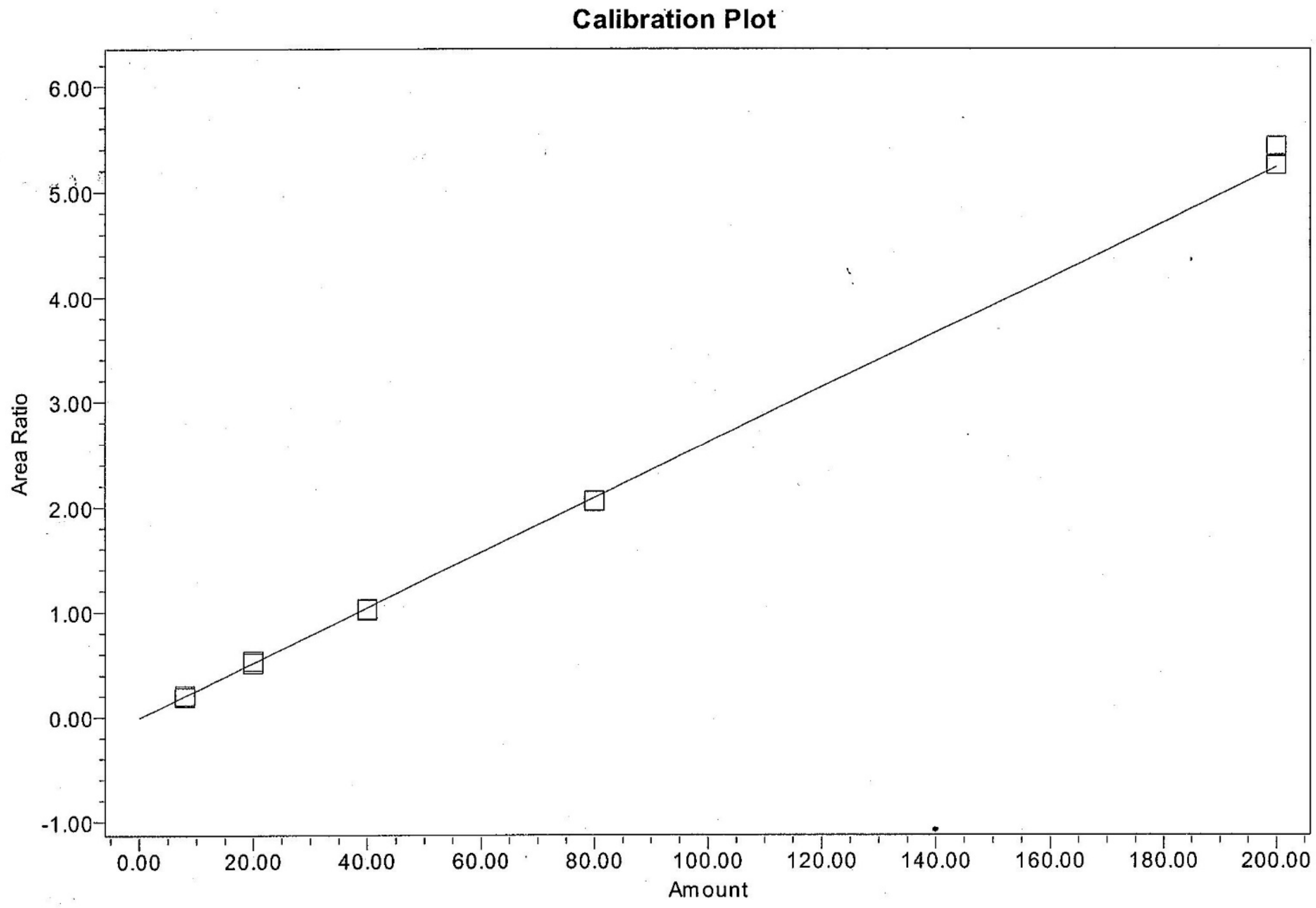

(b)

Figure S1. Calibration plots for plasma (a); and tissue matrices (b). Calibration plots for all tissue matricies (liver, kidney, muscle and fat) were performed similarly.

(a)

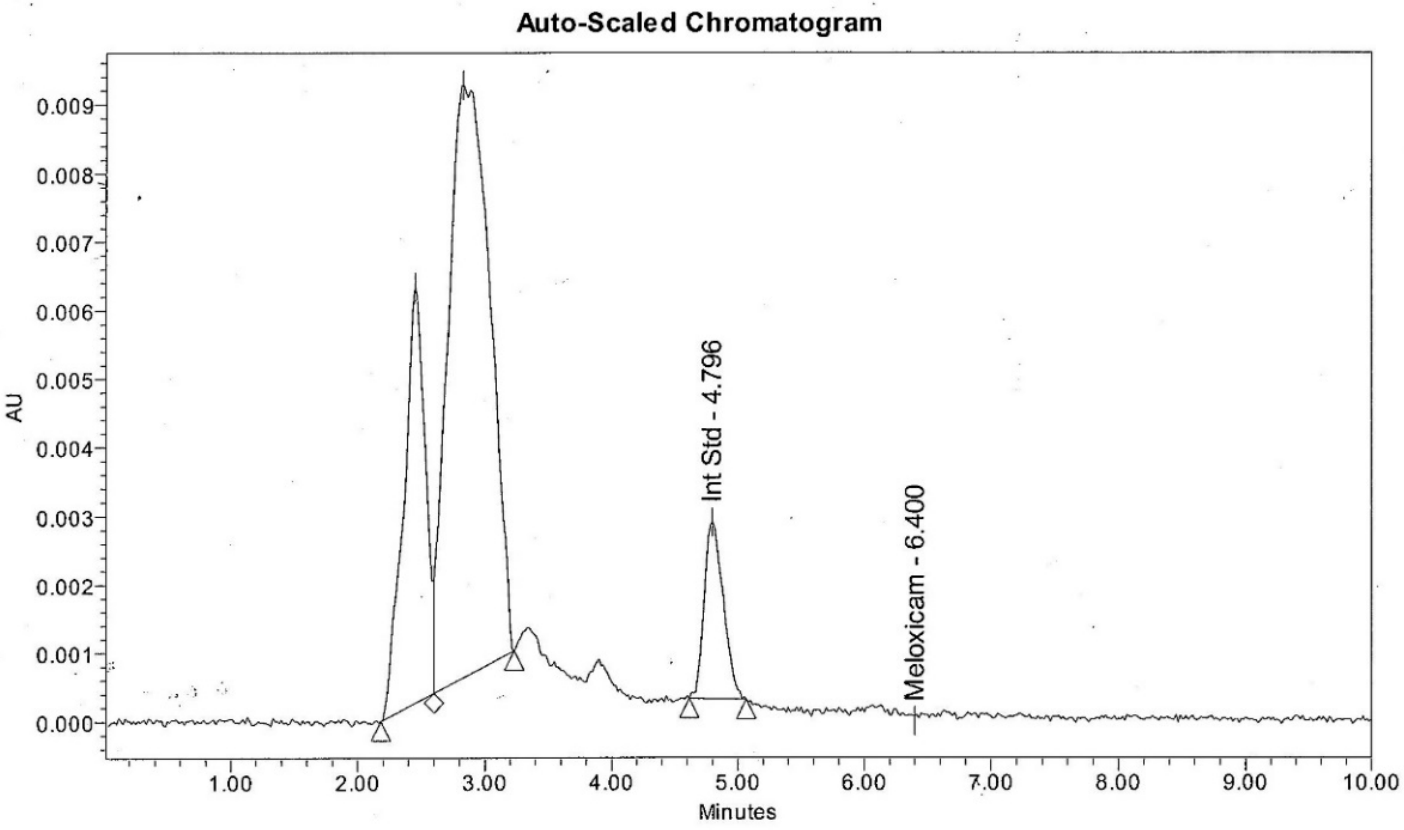
Auto-Scaled Chromatogram
AU
0.009
0.008
0.007
0.006
0.005
0.004
0.003
0.002
0.001
0.000
Int Std - 4.796
Meloxicam - 6.400
1.00
2.00
3.00
4.00
5.00
6.00
7.00
8.00
9.00
10.00
Minutes

(b)

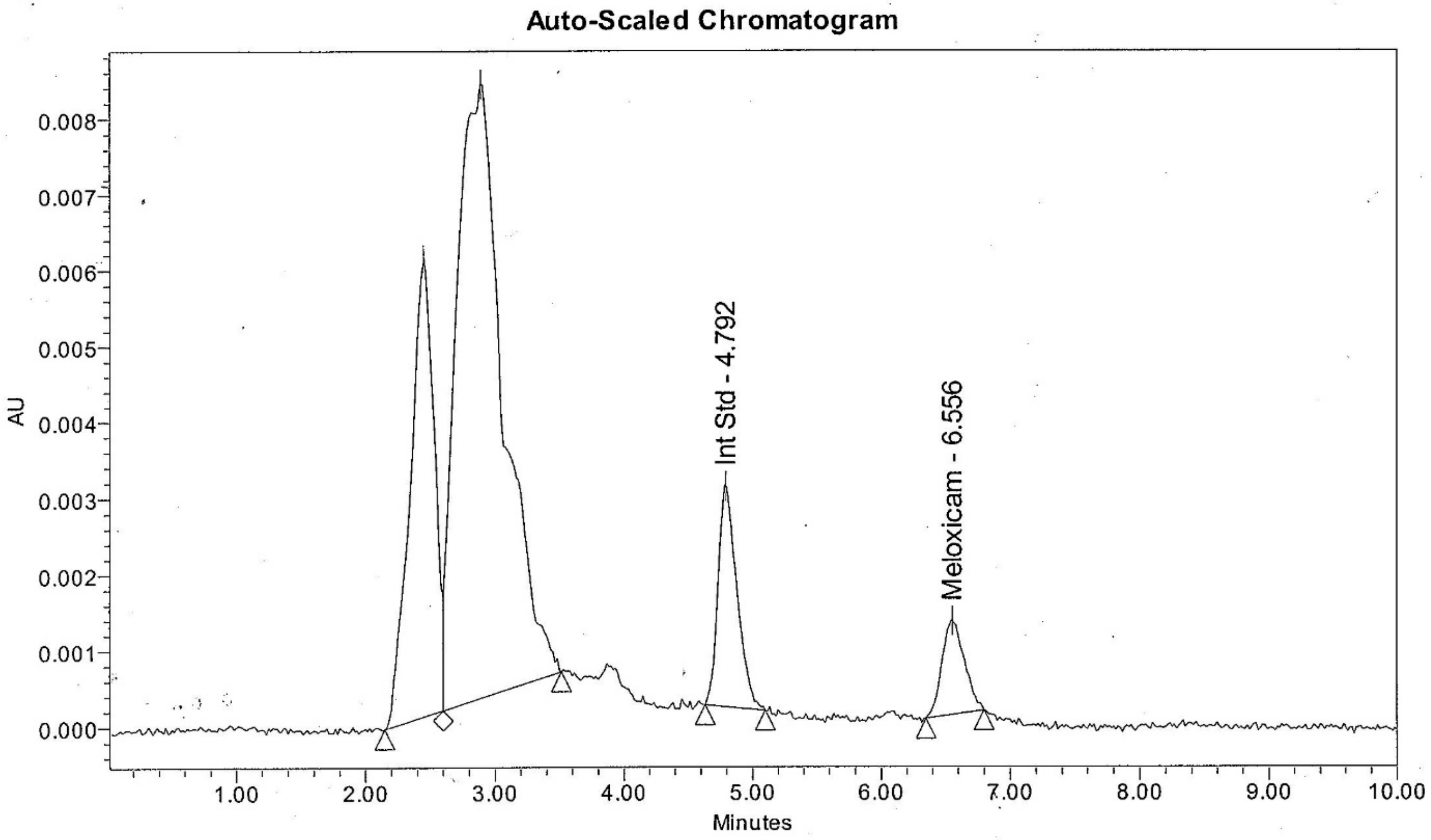
Auto-Scaled Chromatogram
AU
0.008
0.007
0.006
0.005
0.004
0.003
0.002
0.001
0.000
Int Std - 4.792
Meloxicam - 6.556
1.00
2.00
3.00
4.00
5.00
6.00
7.00
8.00
9.00
10.00
Minutes

(c)

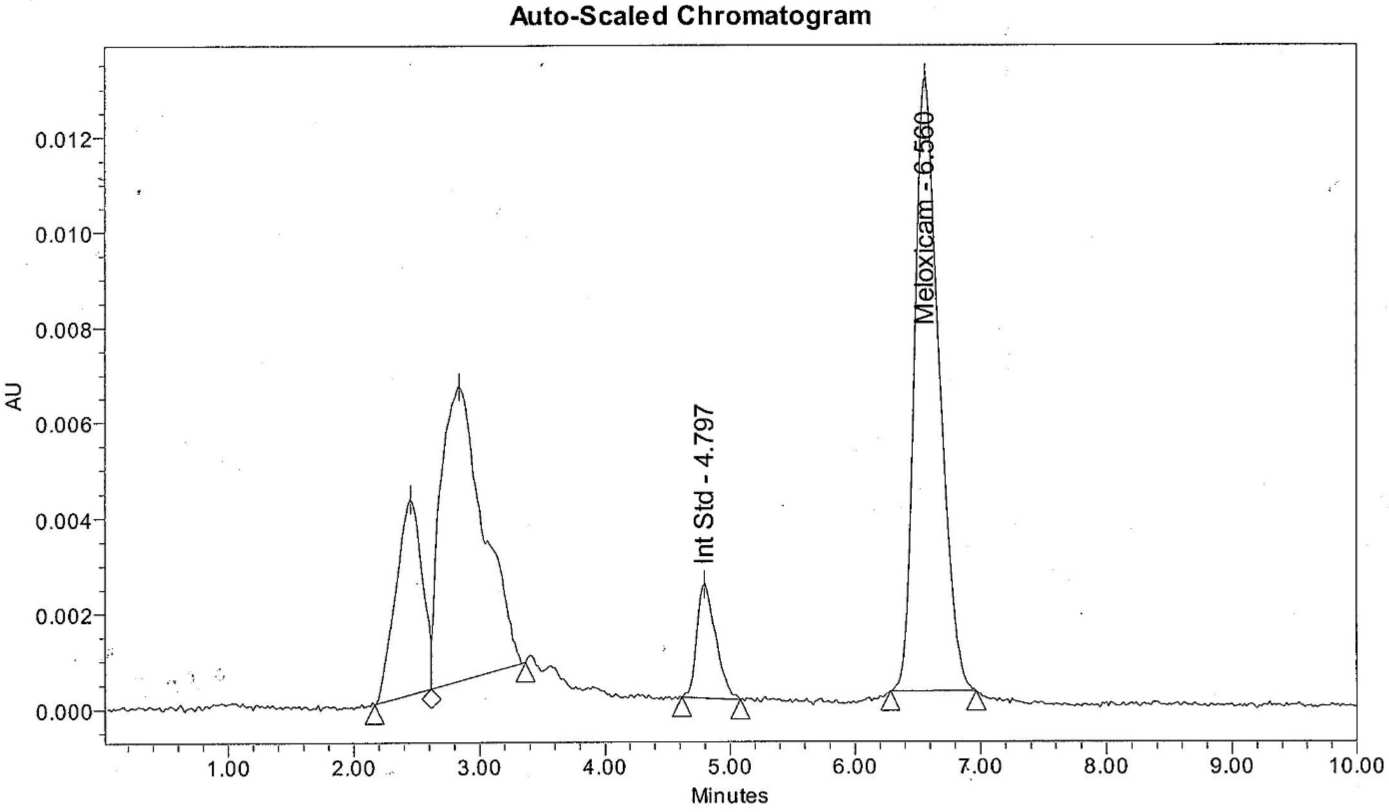

Figure S2: Chromatograms for analysis of meloxicam in sheep plasma. (a) chromatogram of a 0 µg/mL quality control sample; (b) chromatogram of a 0.02 µg/mL quality control sample; (c) chromatogram of an incurred sample.

(a)

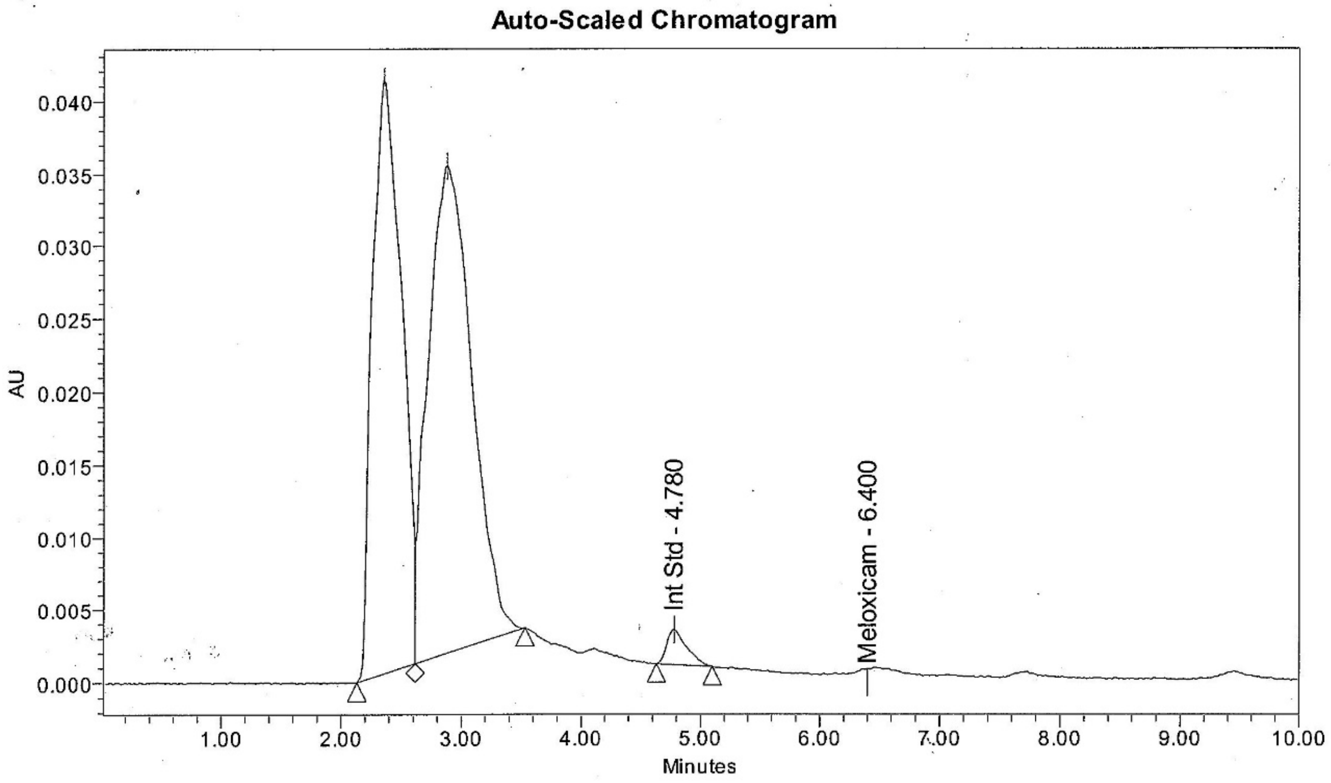
Auto-Scaled Chromatogram
AU
0.040
0.035
0.030
0.025
0.020
0.015
0.010
0.005
0.000
Int Std - 4.780
Meloxicam - 6.400
1.00
2.00
3.00
4.00
5.00
6.00
7.00
8.00
9.00
10.00
Minutes

(b)

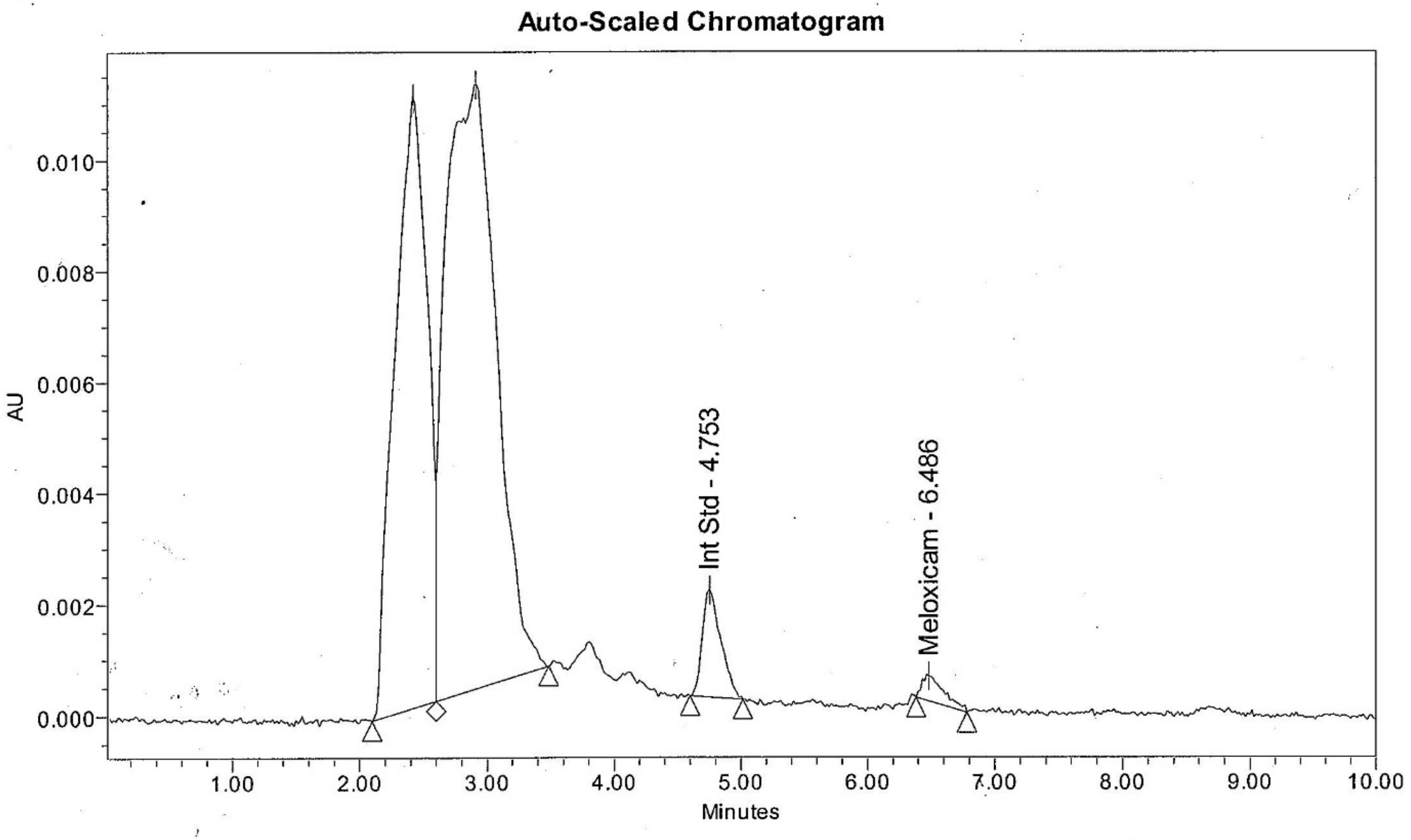
Auto-Scaled Chromatogram
AU
0.010
0.008
0.006
0.004
0.002
0.000
Int Std - 4.753
Meloxicam - 6.486
1.00
2.00
3.00
4.00
5.00
6.00
7.00
8.00
9.00
10.00
Minutes

(c)

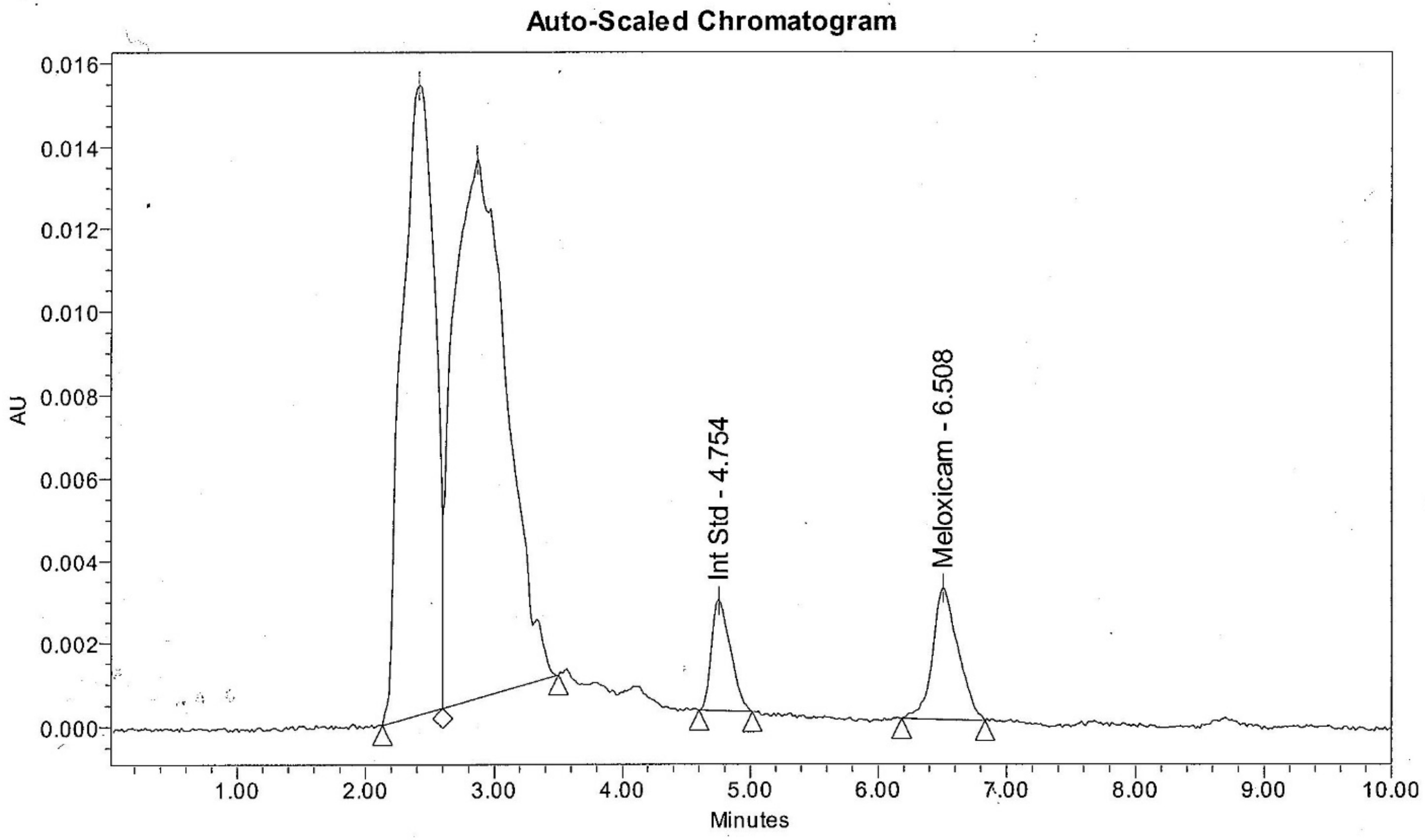

Figure S3: Chromatograms for analysis of meloxicam in sheep liver tissue. (a) chromatogram of a 0 µg/g quality control sample; (b) chromatogram of a 0.02 µg/g quality control sample; (c) chromatogram of an incurred sample.

(a)

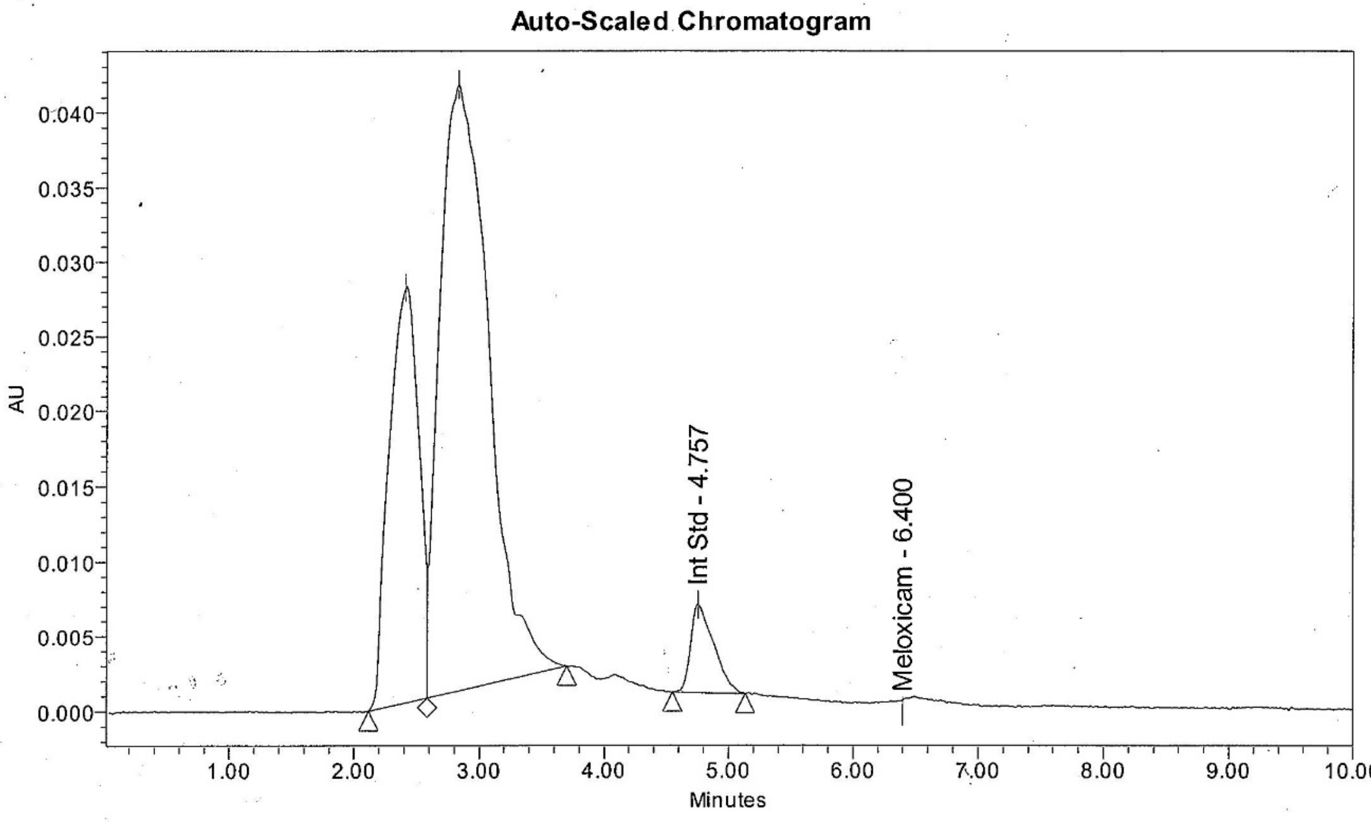
Auto-Scaled Chromatogram
AU
0.040
0.035
0.030
0.025
0.020
0.015
0.010
0.005
0.000
Int Std - 4.757
Meloxicam - 6.400
1.00
2.00
3.00
4.00
5.00
6.00
7.00
8.00
9.00
10.00
Minutes

(b)

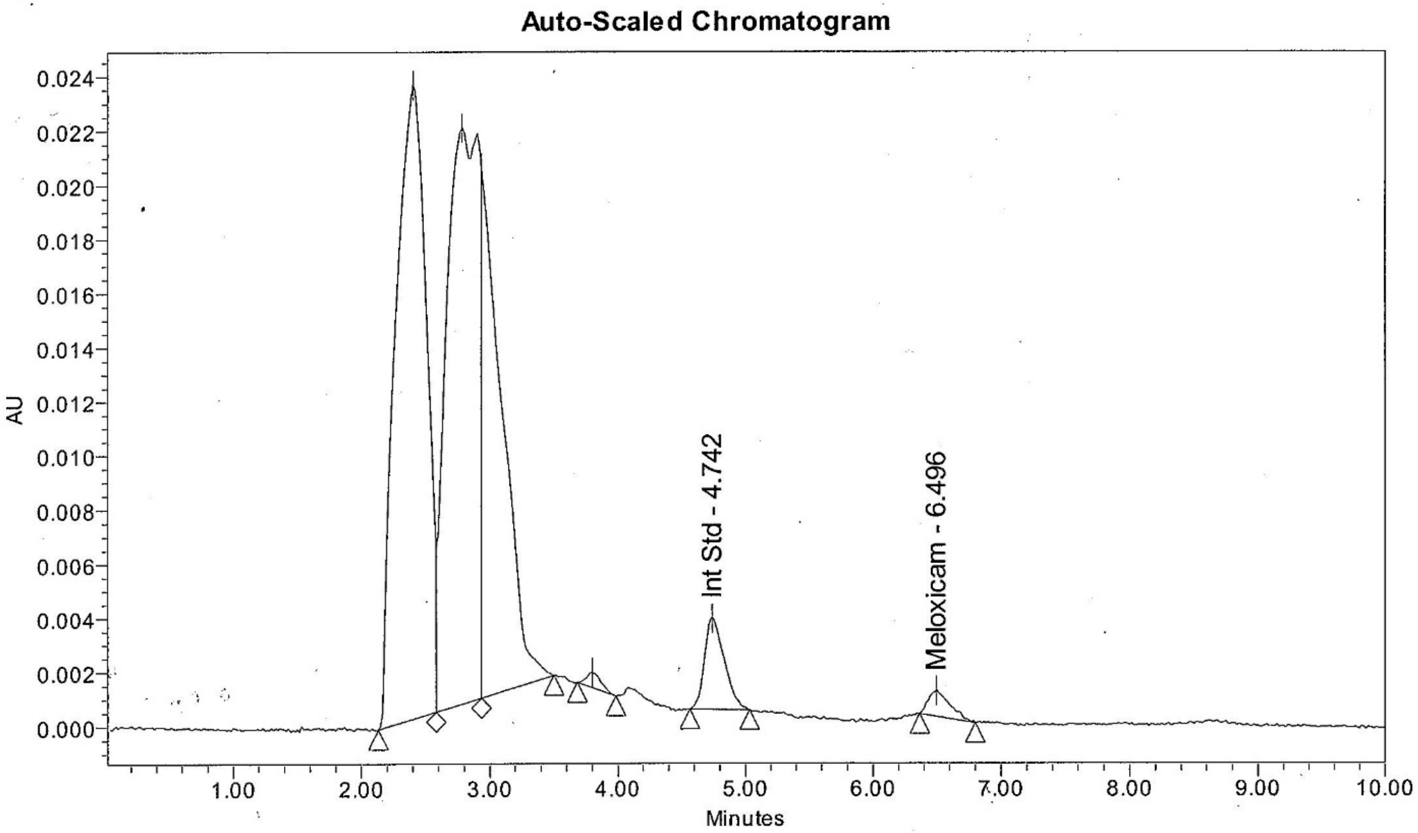
Auto-Scaled Chromatogram
AU
0.024
0.022
0.020
0.018
0.016
0.014
0.012
0.010
0.008
0.006
0.004
0.002
0.000
Int Std - 4.742
Meloxicam - 6.496
1.00
2.00
3.00
4.00
5.00
6.00
7.00
8.00
9.00
10.00
Minutes

(c)

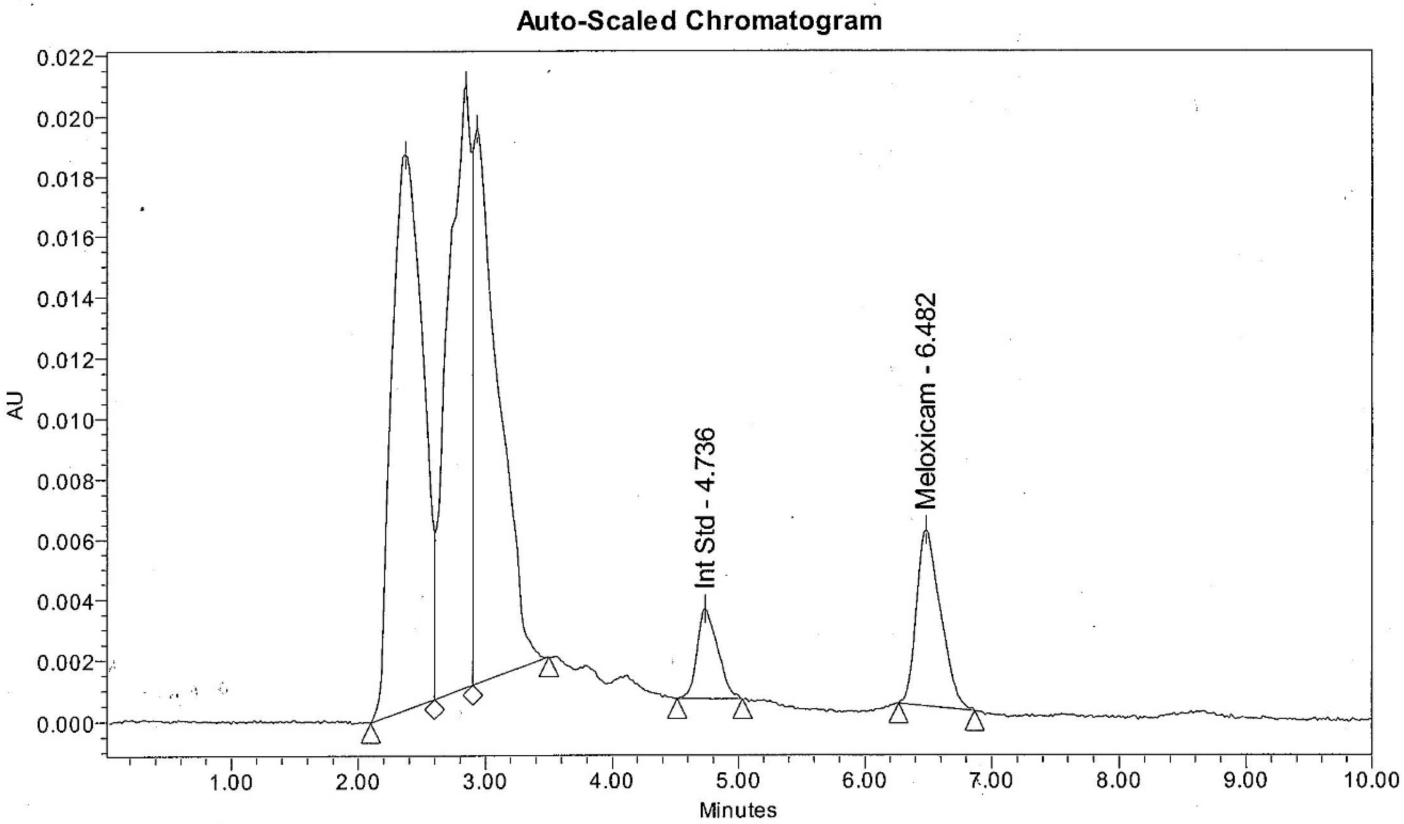

Figure S4: Chromatograms for analysis of meloxicam in sheep kidney tissue. (a) chromatogram of a 0 µg/g quality control sample; (b) chromatogram of a 0.02 µg/g quality control sample; (c) chromatogram of an incurred sample.

(a)

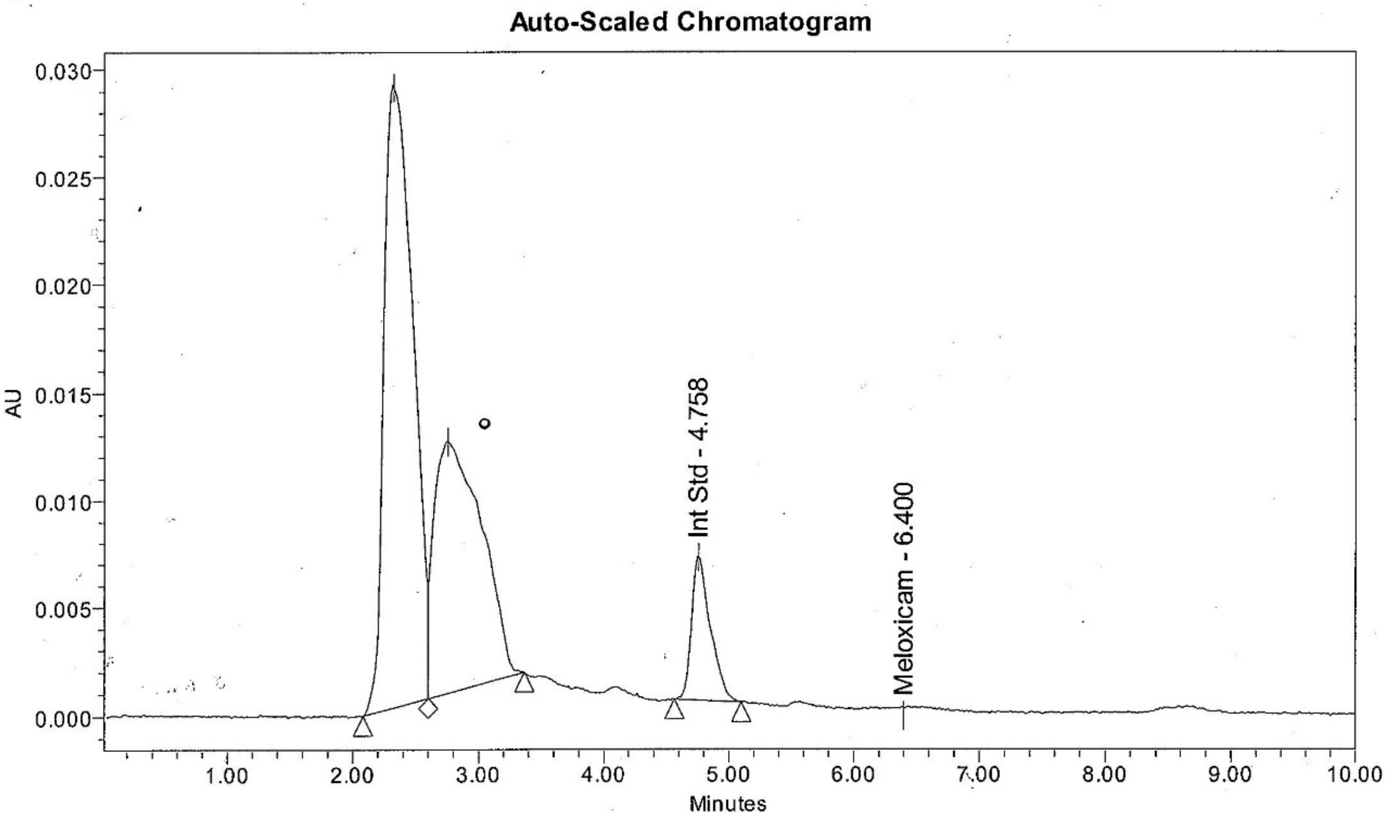

Auto-Scaled Chromatogram
AU
0.030
0.025
0.020
0.015
0.010
0.005
0.000
Int Std - 4.758
Meloxicam - 6.400
1.00
2.00
3.00
4.00
5.00
6.00
7.00
8.00
9.00
10.00
Minutes

b)

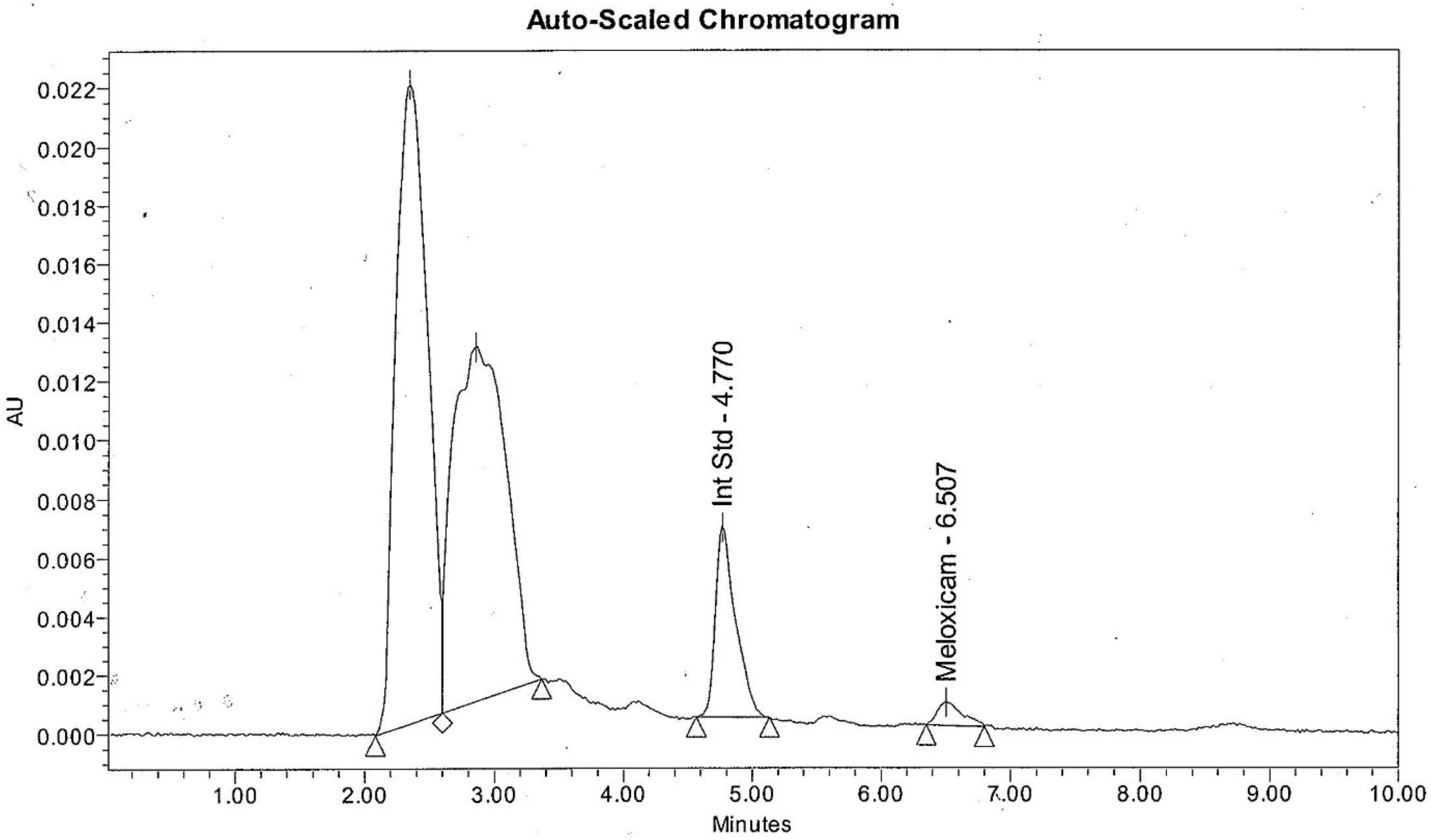
Auto-Scaled Chromatogram
AU
0.022
0.020
0.018
0.016
0.014
0.012
0.010
0.008
0.006
0.004
0.002
0.000
Int Std - 4.770
Meloxicam - 6.507
1.00
2.00
3.00
4.00
5.00
6.00
7.00
8.00
9.00
10.00
Minutes

(c)

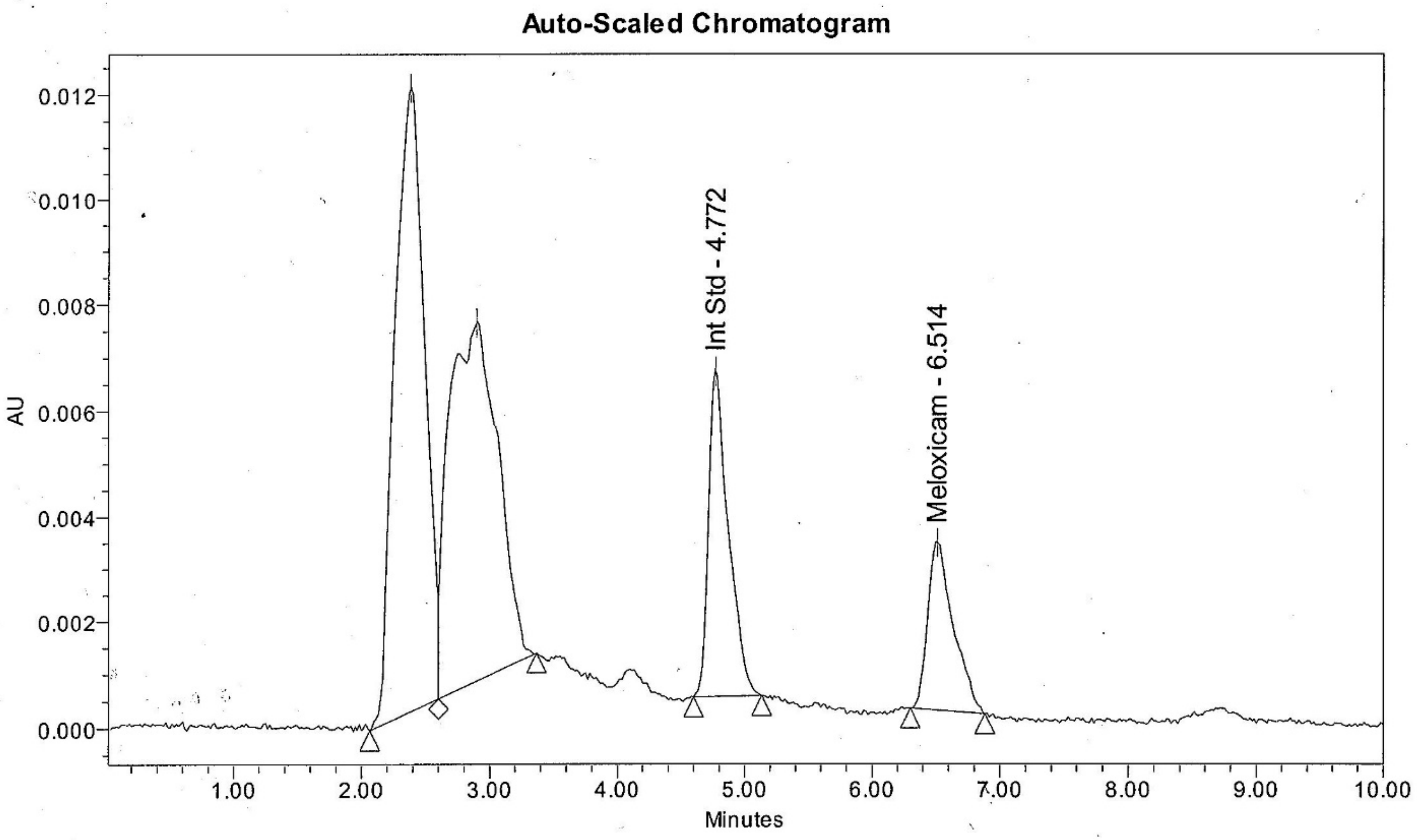

Figure S5: Chromatograms for analysis of meloxicam in sheep muscle tissue. (a) chromatogram of a 0 µg/g quality control sample; (b) chromatogram of a 0.02 µg/g quality control sample; (c) chromatogram of an incurred sample.

(a)

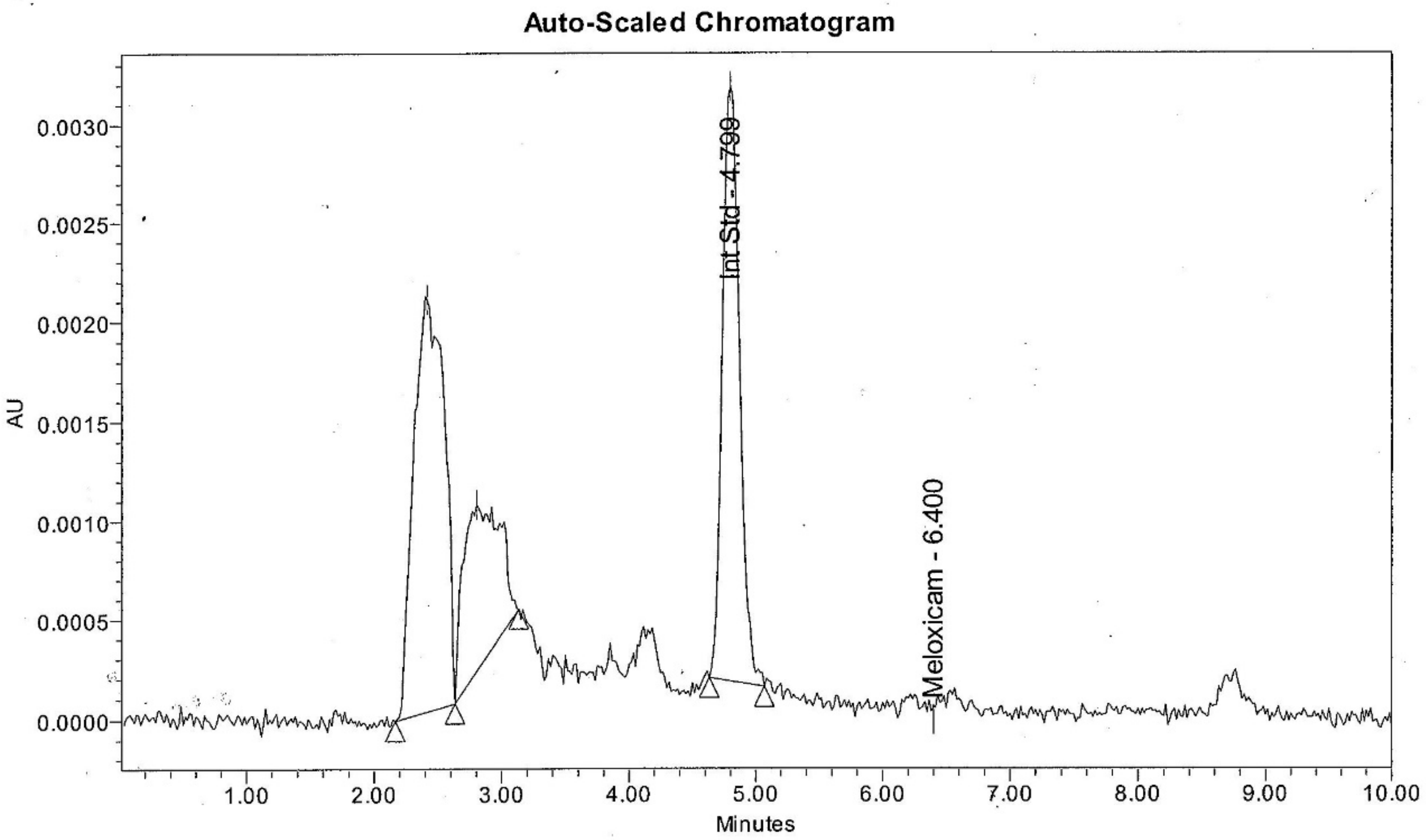

Auto-Scaled Chromatogram
AU
0.0030
0.0025
0.0020
0.0015
0.0010
0.0005
0.0000
Int Std - 4.799
Meloxicam - 6.400
1.00
2.00
3.00
4.00
5.00
6.00
7.00
8.00
9.00
10.00
Minutes

(b)

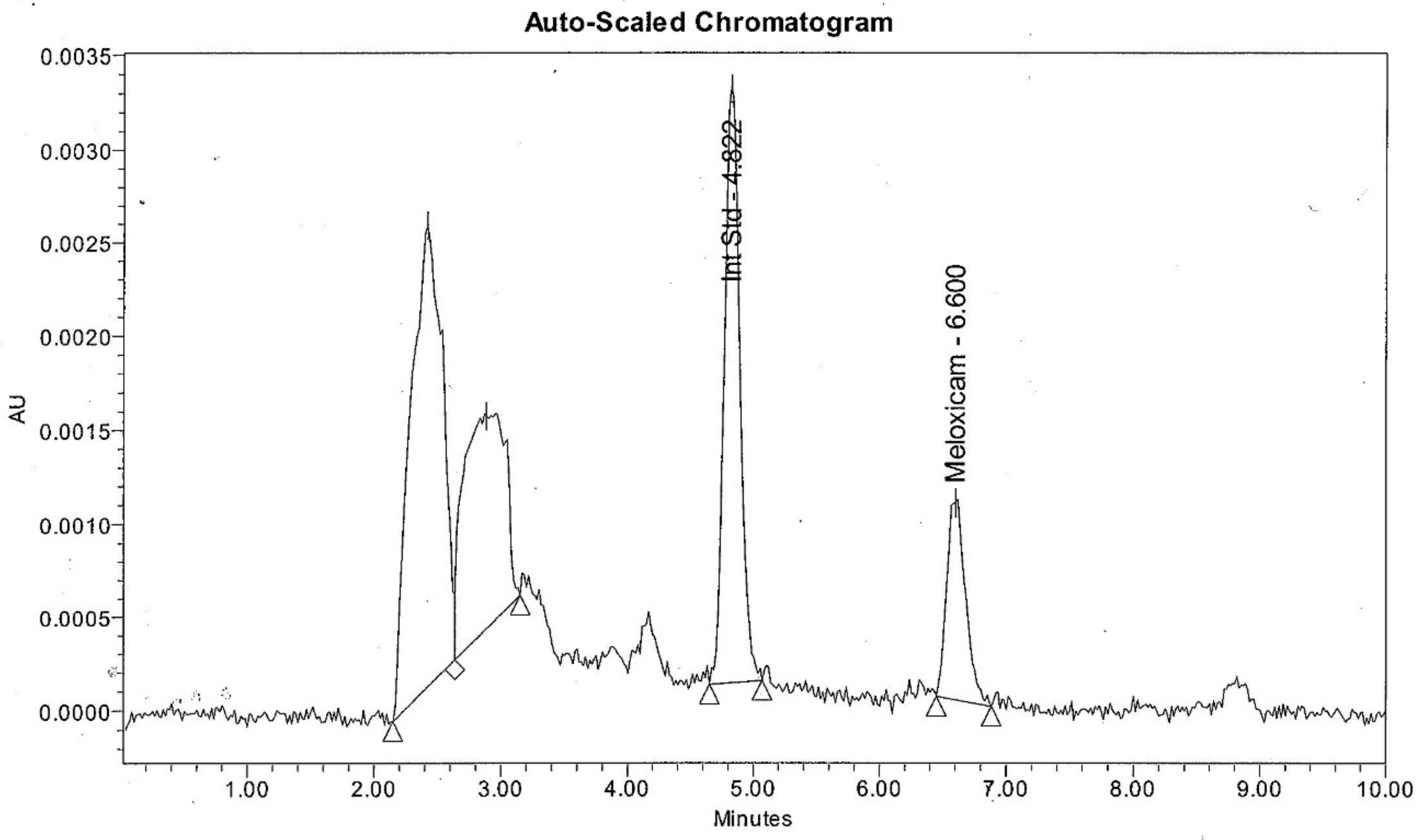
Auto-Scaled Chromatogram
0.0035
0.0030
0.0025
0.0020
0.0015
0.0010
0.0005
0.0000
AU
Int Std - 4.822
Meloxicam - 6.600
1.00
2.00
3.00
4.00
5.00
6.00
7.00
8.00
9.00
10.00
Minutes

(c)

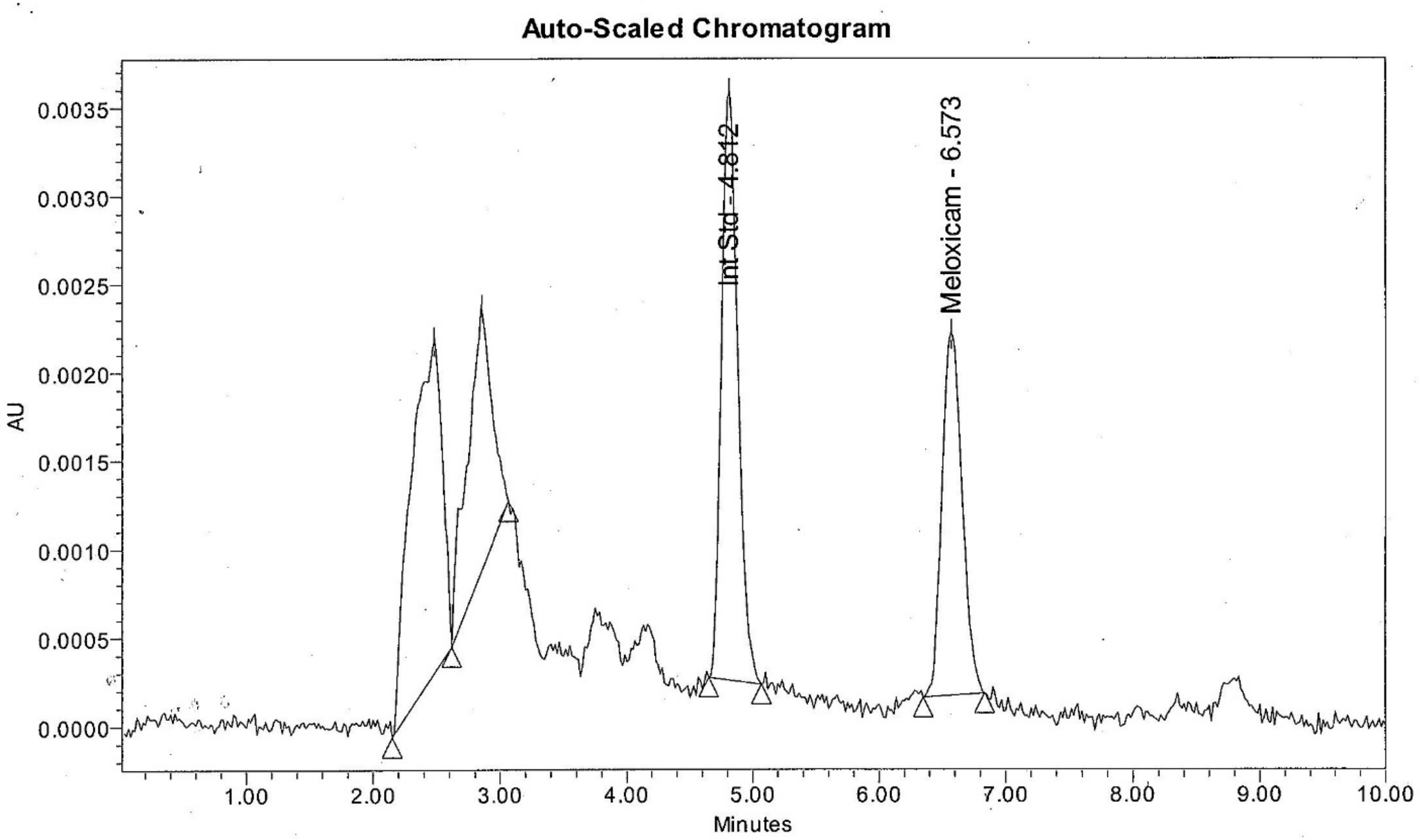

Figure S6: Chromatograms for analysis of meloxicam in sheep fat tissue. (a) chromatogram of a 0 µg/g quality control sample; (b) chromatogram of a 0.02 µg/g quality control sample; (c) chromatogram of an incurred sample.
